# The *Salmonella* T3SS1 effector IpaJ is regulated by ItrA and inhibits the MAPK signaling pathway

**DOI:** 10.1371/journal.ppat.1011005

**Published:** 2022-12-07

**Authors:** Chao Yin, Jiaojie Gu, Dan Gu, Zhenyu Wang, Ruoyun Ji, Xinan Jiao, Qiuchun Li

**Affiliations:** 1 Key Laboratory of Prevention and Control of Biological Hazard Factors (Animal Origin) for Agri-food Safety and Quality, Ministry of Agriculture of China, Yangzhou University, Yangzhou, Jiangsu, China; 2 Jiangsu Key Lab of Zoonosis/Jiangsu Co-Innovation Center for Prevention and Control of Important Animal Infectious Diseases and Zoonoses, Yangzhou University, Yangzhou, Jiangsu, China; 3 Joint International Research Laboratory of Agriculture and Agri-Product Safety, Yangzhou University, Yangzhou, Jiangsu, China; University of Toronto, CANADA

## Abstract

Invasion plasmid antigen J (IpaJ) is a protein with cysteine protease activity that is present in *Salmonella* and *Shigella* species. *Salmonella enterica* serovar Pullorum uses IpaJ to inhibit the NF-κB pathway and the subsequent inflammatory response, resulting in bacterial survival in host macrophages. In the present study, we performed a DNA pull-down assay and EMSA and identified ItrA, a new DeoR family transcriptional regulator that could control the expression of IpaJ by directly binding to the promoter of *ipaJ*. The deletion of *itrA* inhibited the transcription of *ipaJ* in *Salmonella*. Tn-Seq revealed that two regulators of *Salmonella* pathogenicity island 1 (SPI-1), namely HilA and HilD, regulated the secretion of IpaJ. The deletion of *hilA*, *hilD* or SPI-1 inhibited the secretion of IpaJ in both cultured medium and *Salmonella*-infected cells. In contrast, the strain with the deletion of *ssrB* (an SPI-2 regulator-encoding gene) displayed normal IpaJ secretion, indicating that IpaJ is an effector of the SPI-1-encoded type III secretion system (T3SS1). To further demonstrate the role of IpaJ in host cells, we performed quantitative phosphoproteomics and compared the fold changes in signaling molecules in HeLa cells infected with wild-type *S*. Pullorum C79-13 with those in HeLa cells infected with the *ipaJ*-deleted strain C79-13ΔpSPI12. Both phosphoproteomics and Western blot analyses revealed that p-MEK and p-ERK molecules were increased in C79-13ΔpSPI12- and C79-13ΔpSPI12-p*ipaJ*(C45A)-infected cells; and Co-IP assays demonstrated that IpaJ interacts with Ras to reduce its ubiquitination, indicating that IpaJ can inhibit the activation of the MAPK signaling pathway.

## Author summary

As a cysteine protease, IpaJ present in both *Salmonella* and *Shigella* is delivered into host cells to display its activity. However, the expression and secretion of IpaJ remains unclear. Here, we identified a novel DeoR family transcriptional regulator ItrA controlling the transcription of *ipaJ*, and demonstrated that the expressed IpaJ is an effector of T3SS1. IpaJ can be secreted into culture supernatant *in vitro* and host cell *in vivo* dependent on T3SS1. After injection into host cells, IpaJ can not only inhibit the NF-κB pathway and the subsequent inflammatory response through by suppressing IκBα ubiquitination, but also inhibit the activation of the MAPK signaling pathway through downregulation of p-MEK and p-ERK molecules. In summary, we revealed the molecular mechanism of IpaJ expression and secretion in *Salmonella*, and demonstrated its role in inhibiting host MAPK signaling pathway.

## Introduction

*Salmonella enterica* is a facultative intracellular pathogen consisting of more than 2600 serotypes. *Salmonella* infects multiple hosts by using various virulence factors that promote invasiveness and intracellular survival [1,2]. As *Salmonella* infection is mainly caused by the oral ingestion of contaminated food or water, a critical part of infection process is that many genes located on *Salmonella* pathogenicity islands (SPIs) are involved in intestinal penetration and host cell invasion [3]. SPI-1 and SPI-2 were acquired at different evolutionary times and play key roles in *Salmonella* virulence. Both SPI-1 and SPI-2 encode type III secretion systems (T3SSs), effector proteins, chaperones, and transcriptional regulators that control the expression of genes located on SPIs [4]. These virulence factors and effectors that are delivered into the host cell at different stages of infection trigger cellular responses through protein–protein interactions (PPIs) with host cell proteins, resulting in bacterial invasion and replication in host cells [5].

*Salmonella* and other pathogens carrying T3SSs have similar abilities to target many cellular pathways. Some effectors show an antagonistic effect on a specific pathway at different stages of infection [6]. The expression of SopE/SopE2 promotes the actin-based plasma membrane (PM) ruffles required for bacterial internalization but induces MAPK activation and proinflammatory responses [7]. In contrast, the expression of many other effectors (AvrA, SseL, and SptP) can inhibit the critical NF-κB and MAPK signaling cascades to prevent or reduce inflammatory responses and protect bacteria from host immune system attacks. SptP, an SPI-1 effector with GAP activity, downregulates ERK activation and prevents the formation of proinflammatory signaling complexes by dephosphorylating vimentin and cytokeratins [8,9]. AvrA acetyltransferase mediates the suppression of host immune and apoptotic defenses via JNK pathway blockade [10]. Moreover, AvrA has deubiquitinase activity, which can dampen MAPK and NF-κB signaling pathways and facilitate the survival of *Salmonella* in host cells [11,12].

IpaJ, a *Shigella flexneri* effector protein with cysteine protease activity, cleaves an array of N-myristoylated proteins involved in cell growth, signal transduction, auto-phagosome maturation, and organelle function [13]. Our previous study confirmed that IpaJ is also present in *Salmonella* as a specific virulence factor [14–16]. IpaJ plays a role as an important anti-inflammatory protein in *S*. Pullorum infection by inhibiting the NF-κB pathway and the subsequent inflammatory response [15,17]. However, a systemic view of the interactions between IpaJ and host proteins is required to reveal the molecular mechanism underlying its anti-inflammatory effect. In addition, details regarding the expression and secretion of IpaJ are unknown, which hamper us from further understanding the expression mechanism of *ipaJ* as a virulence factor at the molecular level.

In the present study, we performed Tn-Seq to screen for regulators that control the expression and secretion of IpaJ. HilA and HilD mainly regulated the secretion of IpaJ into host cells in a T3SS1-dependent manner, indicating that IpaJ is an effector of T3SS1. A DNA pull-down assay further identified IpaJ transcription regulator A (ItrA), a protein belonging to DeoR family transcriptional regulators that could directly bind to the *ipaJ* promoter. In addition, we used a label-free quantitative phosphoproteomic approach and demonstrated that IpaJ could downregulate the phosphorylation of ERK and FAK molecules to inhibit the activation of MAPK pathways and the subsequent inflammatory response in *Salmonella*-infected HeLa cells.

## Results

### Tn-Seq-based screening reveals the regulators of *ipaJ* expression

**Fig 1:**
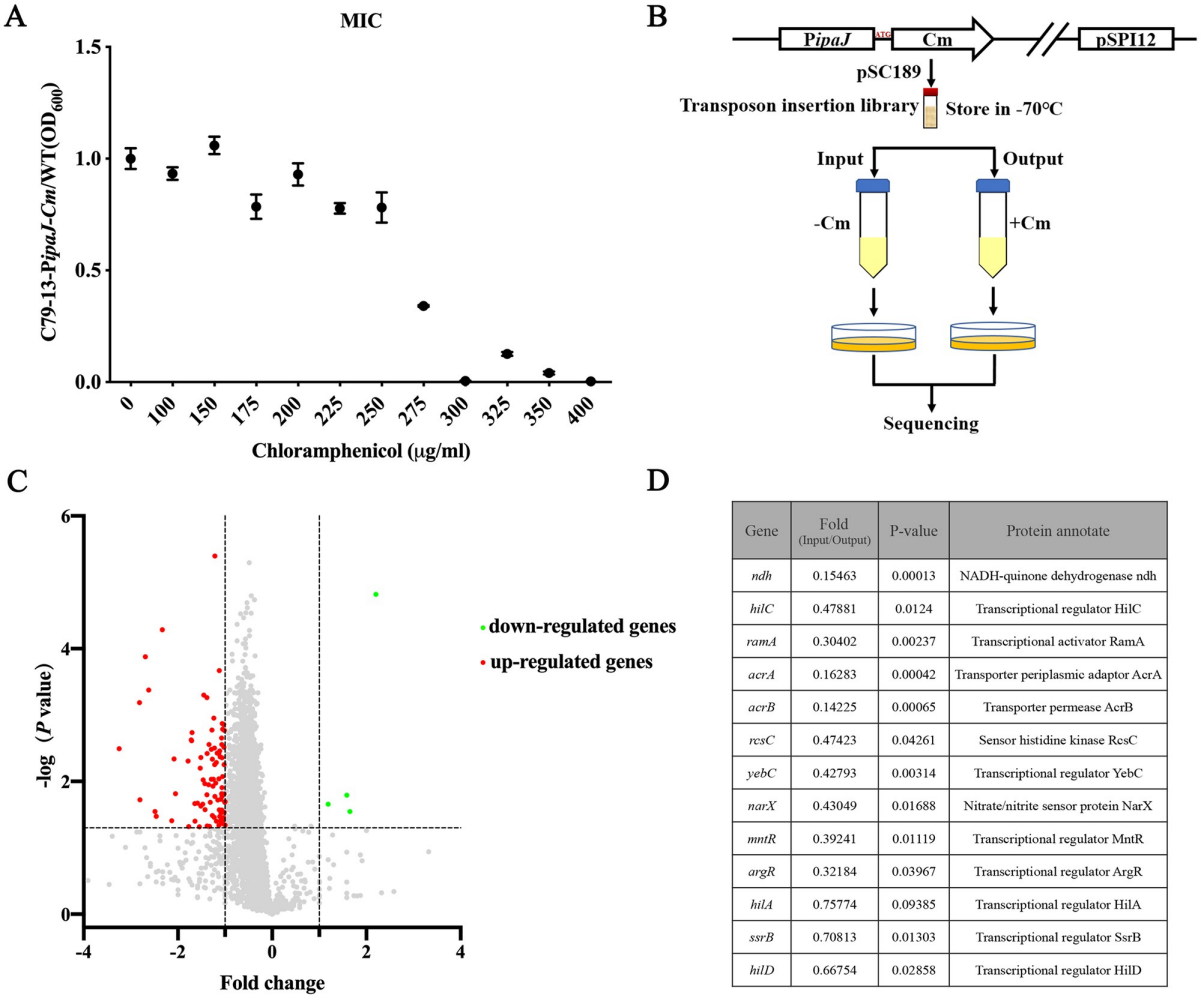
Identification of factors regulating the expression of IpaJ using Tn-Seq. (**A**) Relative growth of the C79-13-P*ipaJ*-*Cm* vs. WT strains grown in LB medium with serially diluted concentrations of Cm. (**B**) Schematic representation of the Tn-Seq strategy used to identify regulators controlling *ipaJ* expression and secretion. A transposon library was created in the *S*. Pullorum C79-13 strain by inserting a Cm resistance gene downstream of the *ipaJ* start codon. The library was grown in LB medium in the presence or absence of Cm. The sites and abundance of transposon insertions under these two conditions were compared. (**C**) Volcano plot showing the output/input FC in reads of genes, as revealed by Tn-Seq analysis. The genes of interest were highlighted with cut-offs of |log2(FC)| > 1 and *P*-value < 0.05. (**D**) Thirteen regulatory factors potentially promoted the expression of IpaJ.

As IpaJ is a key anti-inflammatory protein in *S*. Pullorum and *ipaJ* is located in the pSPI12 plasmid [15], a Tn-Seq-based screening method was devised to identify upstream regulators that can affect *ipaJ* expression. Initially, the reporter strain of C79-13-P*ipaJ*-*cm* was constructed by fusing a promoterless Cm resistance gene to the *ipaJ* promoter P*ipaJ*. Following this, a high-density Himar transposon insertion library was created in C79-13-P*ipaJ*-*cm*; the library was grown in LB medium in the absence (input) or presence (output) of Cm (Fig 1A). Finally, high-throughput sequencing was performed to identify the transposon-inserted sites and enumerate the frequency of insertions in the input and output libraries. Mutants present in the input library but absent in the output library were considered to have critical insertions in the loci for IpaJ expression. In contrast, mutants present at a higher frequency in the output library were considered to represent transposon-inserted genes that could potentially inhibit IpaJ expression. To estimate the optimal concentration of Cm to be used for screening the library, we assessed the maximum inhibitory concentrations (MICs) of C79-13-P*ipaJ*-*cm*. The strain typically grew at Cm concentrations lower than 275 μg/ml; thus, 250 μg/ml Cm was added to LB medium for screening the transposon library (Fig 1B).

We compared the transposon distribution profiles in the input and output libraries using the Con-ARTIST pipeline to identify genes that were either under- or over-represented (|log2(FC)| > 1 and P < 0.05) in the output library. In total, 94 genes with a diminished abundance of insertions were detected in the Cm-containing LB medium cultures, suggesting that these genes can promote IpaJ expression (Fig 1C and S3 Table). Among the 94 mutants, 18 were identified to show significant differences in IpaJ expression compared to the WT strain. Gene function prediction analysis showed that seven out of the 18 genes encoded transcriptional regulators. Of the seven regulators, HilC was involved in the positive regulation of HilD, in addition to subsequent SPI-1/SPI-2 regulators, such as *hilA*, *hilD*, and *ssrB*, which also had fewer insertions in the output library than in the output one. To validate a subset of the screening hits, 10 genes were selected for constructing insertion mutants; these included a transcriptional regulatory cascade consisting of *hilA*, *hilD*, and *ssrB*, which could positively control the expression of SPI-1 and SPI-2 genes (Fig 1D).

### IpaJ is an effector of SPI-1/T3SS1

When *Salmonella* is grown in LB medium, SPI-1 and SPI-2 regulons are sequentially activated at different stages of the stationary phase [3]. To assess the expression profiles of IpaJ at different growth stages, we examined the expression of IpaJ and HilA by Western blot analysis in the C79-13 strain. The expression of the SPI-1 regulator HilA was detected at 4 h of growth, was more evident at 6 h, and declined at the late stationary phase (S1 Fig). On the other hand, the expression of IpaJ was detected at 6 h of growth, reached the maximum levels between 8 h and 10 h, and dramatically decreased at the late stationary phase, indicating that the growth-dependent expression of IpaJ can be potentially attributed to the activation of HilA.

**Fig 2:**
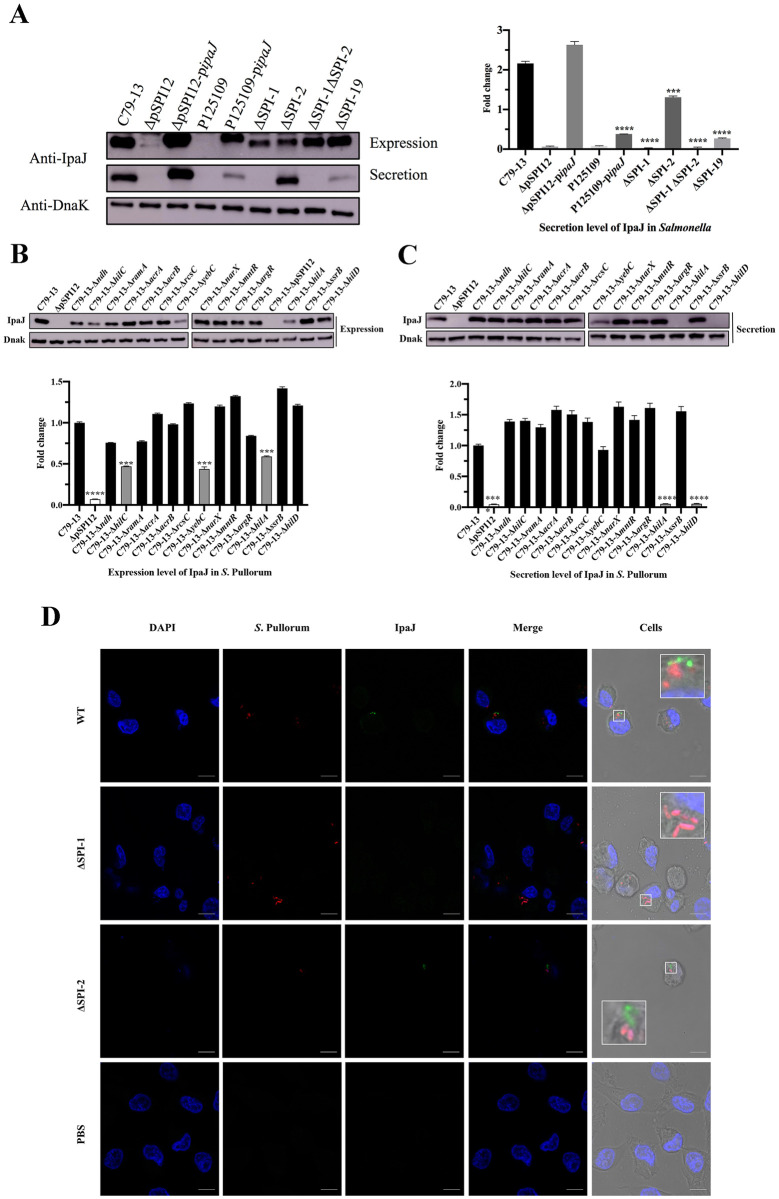
SPI-1 regulates the secretion of IpaJ. (**A**) Immunoblotting analysis of secreted IpaJ proteins isolated from cultured LB medium supernatant. The mutants SPI-1 (ΔSPI-1), SPI-2 (ΔSPI-2), SPI-19(ΔSPI-19) or SPI-1 and SPI-2 (ΔSPI-1ΔSPI-2) were used to detect whether the secretion of IpaJ is dependent on SPI-1, SPI-2 or SPI-19. The *ipaJ-*deleted strain (ΔpSPI12) and the WT/Complementary strains (C79-13 and ΔpSPI12-p*ipaJ*) were used as the negative and positive controls, respectively. The *S*. Enteritidis P125109 strain transformed with pBR322-*ipaJ* (P125109-p*ipaJ*) was also used as another positive control for P125109. (**B-C**) Western blot analysis of samples from bacterial cultures grown in LB medium at 37°C with an OD600 value of 0.5. Bacterial cell lysates (**B**) and secreted proteins from the supernatants (**C**) were subjected to Western blot analysis. (**D**) Immunofluorescence microscopic analysis of T3SS-1-dependent translocation of IpaJ. HD-11 cells were infected with the WT strain or ΔSPI-1 or ΔSPI-2 at an MOI of 200. Red fluorescence represents bacteria carrying pRFP plasmid, green fluorescence represents the expression of IpaJ.

*S*. Pullorum ΔSPI-1, ΔSPI-2, and ΔSPI-19 mutants were then subjected to Western blot analysis in order to assess IpaJ expression and secretion levels. Compared with the wild-type (WT) strain, both ΔSPI-1 and ΔSPI-1ΔSPI-2 strains showed decreased expression levels of IpaJ. The protein was not detected in the culture supernatants of both these strains; However, it could be secreted into the supernatants of the ΔSPI-2 strain, indicating the IpaJ secretion is dependent on SPI-1. Transformation of the plasmid p*ipaJ* in *S*. Enteritidis could also induce the expression of IpaJ (Fig 2B); but the deletion of SPI-19 (ΔSPI-19) significantly decreased the secretion of IpaJ, consistent with SPI-19 mutants showing reduced expression/function of SPI-1 (Fig 2B). These findings indicate that IpaJ secretion is dependent on SPI-1/T3SS1, but IpaJ expression is not affected by SPI-1. Western blot analysis was also performed to assess the expression and secretion levels of IpaJ in the WT and mutant strains at the early stationary phase (Fig 2C). The results revealed that deletion of *hilC*, *yebC*, or *hilA* gene decreased the expression of IpaJ and deletion of *hilA* or *hilD* in *S*. Pullorum inhibited the secretion of IpaJ into the supernatant (Fig 2C). In addition, we inserted the ORF of *ipaJ* into the plasmid pBAD33 with an arabinose-inducible promoter P(BAD) and transformed it into the Δ*hilA* and ΔSPI-1 strains. Western blot analysis revealed that arabinose induced high expression of IpaJ, but IpaJ could not be secreted into the supernatant without SPI-1/T3SS1 (S2 Fig). In summary, these findings indicate that IpaJ is an effector of *Salmonella* T3SS1.

To further verify whether IpaJ can be transported into cells through T3SS-1, IpaJ was injected into the host cell for immunofluorescence analysis using the monoclonal antibody 4G6 against IpaJ as the primary antibody. IpaJ was detected in the cytoplasm of WT- and ΔSPI-2-infected HD-11 cells and separated from bacteria of red fluorescence, but IpaJ was not detected in the ΔSPI-1-infected cells (Fig 2D), indicating that IpaJ is an effector of T3SS1 and can be transported into host cells by T3SS1 during *Salmonella* infection. We further cloned *ipaJ* into a pCX340 vector to fuse IpaJ with TEM-1 β-lactamase (IpaJ-TEM-1) and transformed the plasmid into the WT, ΔSPI-1, and ΔSPI-2 strains. IpaJ translocation into HeLa cells infected with *Salmonella* was monitored using a CCF2-AM fluorescence resonance energy transfer (FRET) assay. In WT-pCX34-*ipaJ*- and ΔSPI-2-pCX34-*ipaJ*-infected HeLa cells, IpaJ-TEM-1 was translocated into HeLa cells to cleave CCF2 molecules to produce a blue fluorescence signal. In contrast, inΔSPI-1-pCX34-*ipaJ*-infected cells, intact CCF2 molecules emitted a green fluorescent signal, similar to that in WT-pCX34-infected cells (S3 Fig).

### ItrA is identified as the direct regulator of *ipaJ* using a DNA pull-down assay and EMSA

**Fig 3:**
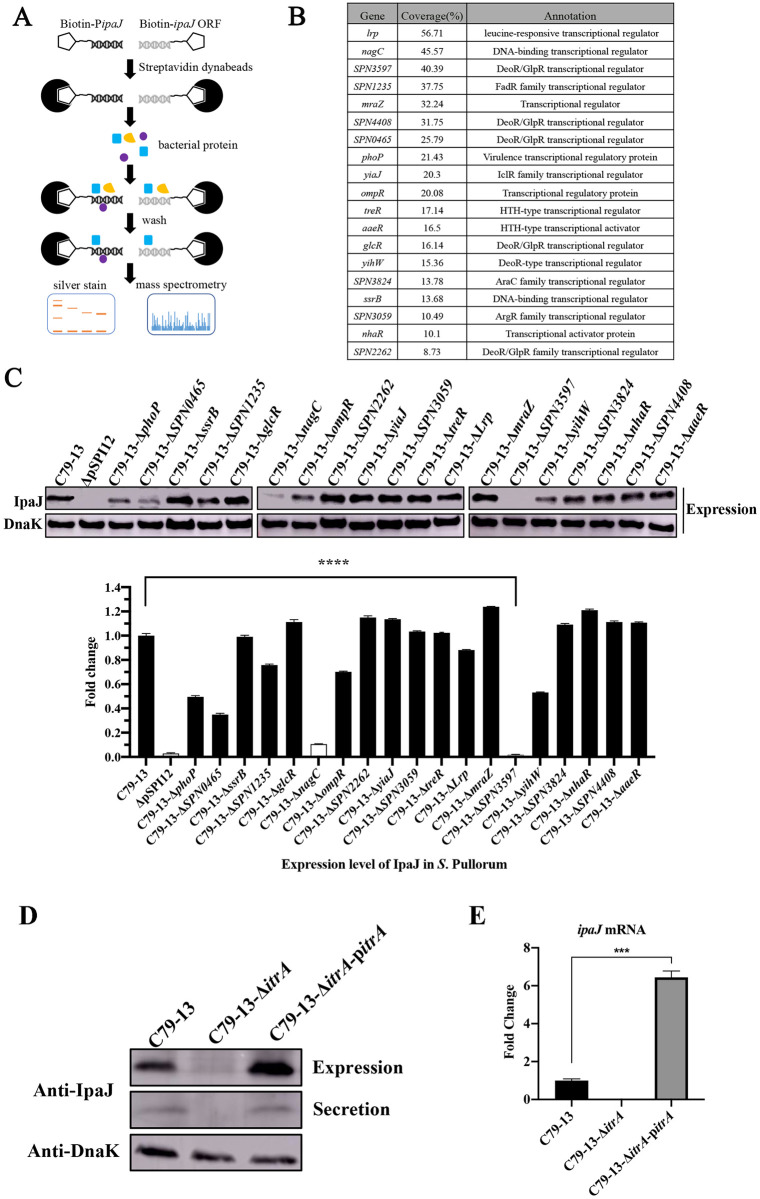
The regulator ItrA promotes *ipaJ* expression. (**A**) Schematic representation of the DNA pull-down assay to screen out the direct regulator binding to the promoter of *ipaJ* (P*ipaJ*). Biotin-P*ipaJ* (sample) and biotin-*ipaJ*orf (negative control) fragments were used as the bait DNA. (**B**). The MS results of identified potential regulators binding to P*ipaJ*. Protein-encoding genes without assigned names were displayed using SPN with a genetic code number. (**C**) Western blot analysis of IpaJ expression in WT and mutant strains cultured in LB medium at 37°C. DnaK was used as the control. Results are representative of three independent experiments. (*) Indicates the significant differences in IpaJ expression levels in different strains vs. the C79-13 group. ****: *P* < 0.0001. (**D**) Western blot analysis of IpaJ expression and secretion in WT, C79-13-Δ*itrA*, and C79-13-Δ*itrA*-p*itrA* strains cultured in LB medium at 37°C. (E) qRT-PCR analysis of *ipaJ* in WT, C79-13-Δ*itrA*, and C79-13-Δ*itrA*-p*itrA* strains cultured in LB medium at 37°C. ***: *P* < 0.001.

To identify direct regulators of *ipaJ*, we performed a DNA pull-down assay using the P*ipaJ* as the bait DNA and cell lysates as the prey proteins. P*ipaJ* labelled with biotin at the 5′ end (biotin-P*ipaJ*) was used as the bait, and the ORF of *ipaJ* labeled with biotin (biotin-*ipaJ*orf) was used as the negative control. After incubation with *S*. Pullorum cell lysates, the complex of streptavidin beads, biotin-P*ipaJ*/biotin-*ipaJ*orf, and the bound putative regulators were eluted with a concentration gradient of NaCl solutions (Fig 3A). Compared with the biotin-*ipaJ*orf group, SDS-PAGE revealed about seven significant bands in the biotin-P*ipaJ* group (S4 Fig). Therefore, the eluted samples were subjected to protein identification using MS (Fig 3B). On comparing the proteins captured by biotin-P*ipaJ* with those captured by biotin-*ipaJ*orf, 35 differential regulators were obtained; most of these belonged to the DeoR/GlpR or AraC family regulators. The proteins with high coverage were the lrp (56.71%), NagC (45.57%), and SPN3597 (40.39%) (S4 Table). To validate the results of the pull-down assay, deletion mutants were constructed for each of the 19 regulatory genes and the expression level of IpaJ was assessed by Western blot analysis (Fig 3C). In contrast to the WT strain, deletion of the *SPN0465* or *nagC* gene significantly decreased the expression of IpaJ, while deletion of the *SPN3597* completely inhibited the expression of IpaJ (Fig 3C), suggesting that SPN3597 is a potential direct regulator of IpaJ. We named the protein SPN3597 as IpaJ transcription regulator A (ItrA). Subsequently, we constructed a complementary strain (C79-13-Δ*itrA*-p*itrA*) by transforming pBR322-*itrA* into C79-13-Δ*itrA*. Both Western blot analysis and qRT-PCR confirmed that the complementary strain had increased IpaJ expression compared with the WT strain (Fig 3D and 3E). These findings indicated that ItrA is involved in the regulation of *ipaJ* transcription and expression.

**Fig 4:**
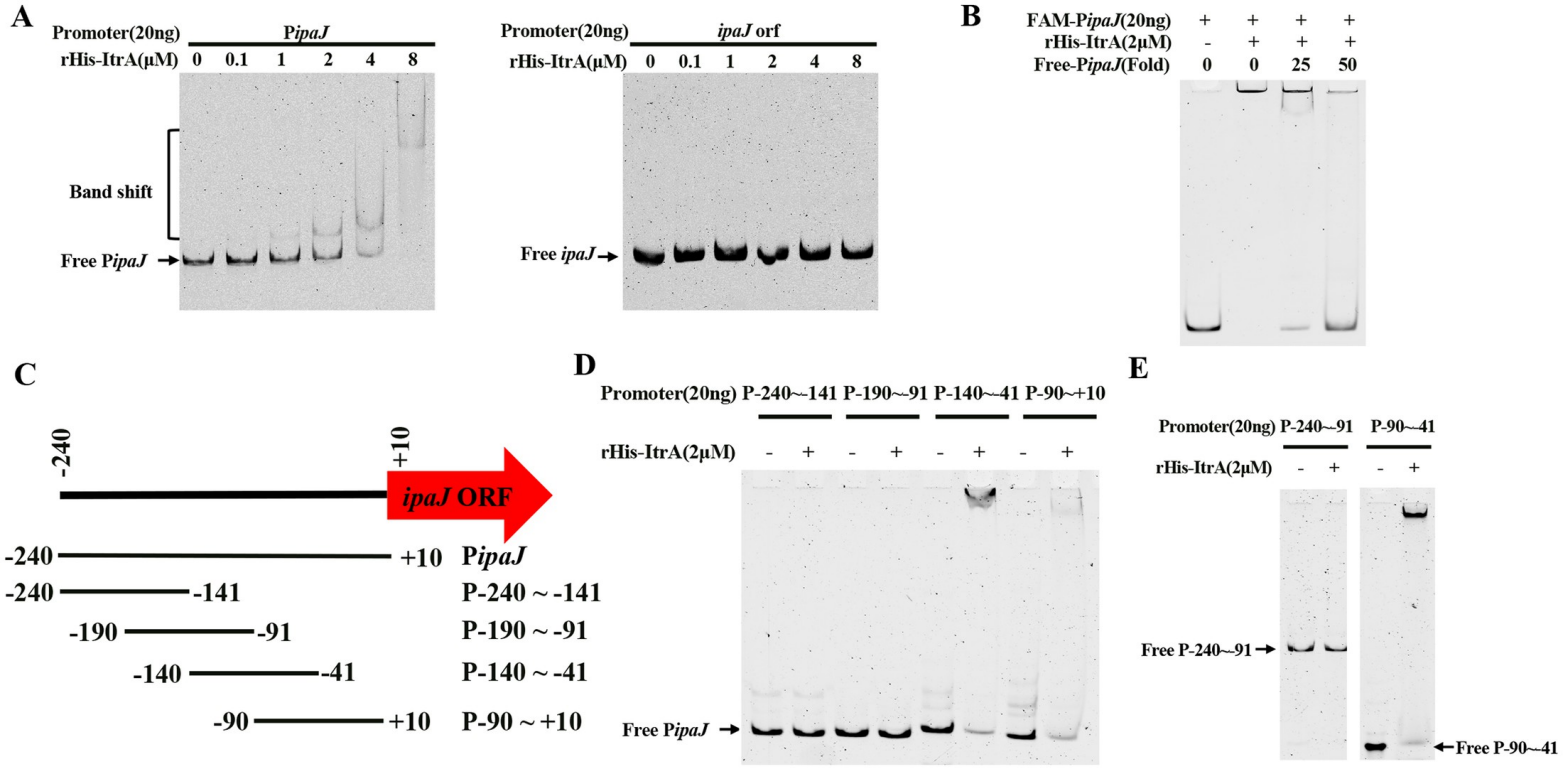
ItrA directly binds to the *ipaJ* promoter region. (**A**) FAM-labeled probes from P*ipaJ* or *ipaJ*orf were used for EMSA with 0, 0.1, 1, 2, 4 or 8 μM of purified rHis-ItrA. (**B**) FAM-labeled probes from P*ipaJ* were incubated with 2μM purified rHis-ItrA in the presence of 25- and 50- fold excess of unlabeled P*ipaJ* competitors. (**C**) Distribution of the four gradient fragments in the P*ipaJ* region. The length of each of the four fragments is 100 bp. (**D)** Four FAM-labelled fragments were subjected to an EMSA assay using 2 μM rHis-ItrA. (**E**) FAM-labeled probes (P-240~-91 and P-90~-41) were used for EMSA assay with 0, 0.1, 1, 2, 4, or 8 μM purified rHis-ItrA.

To determine whether ItrA can directly control the expression of IpaJ, we performed EMSA using purified rHis-ItrA and the P*ipaJ* fragment. The results revealed that the mobility of FAM-P*ipaJ* was reduced with the addition of rHis-ItrA. This shift could be recovered with the addition of non-labelled P*ipaJ* (Fig 4A). However, there was no effect on the shift when the *ipaJ*orf DNA probe was used. Specific binding of rHis-ItrA to the FAM labeled P*ipaJ* was further confirmed by the competitive EMSA (Fig 4B). These results indicate that ItrA can directly bind to the promoter region of *ipaJ*.

To further identify the region containing all the *cis*-acting elements required for ItrA-mediated IpaJ regulation, several fragments of P*ipaJ* of different lengths were constructed (P-240~-141; P-190~-91; P-140~-41; P-90~+10) (Fig 4C). These fragments were then incubated with or without the purified rHis-ItrA. Of the four DNA fragments, the addition of rHis-ItrA to the reaction mixture caused a specific shift in the mobility of the two fragments (P-140~-41; P-90~+10) (Fig 4D), indicating that the binding site was from −90 bp to -41 bp. Therefore, the P-90~-41 fragment was labelled and subjected to EMSA. This shift was observed in the presence of rHis-ItrA (Fig 4E). In addition, the shift of P*ipaJ* was noted in the presence of increasing concentrations of rHis-ItrA (S5 Fig). These results indicated that the 50-bp region (P-90~-41) of P*ipaJ* harbored essential elements that could mediate direct binding to ItrA.

ItrA and HilA play different roles in IpaJ expression and secretion. To determine whether HilA was involved in the transcription of *itrA* and *ipaJ*, we assessed the mRNA levels of *ipaJ* and *itrA* in the Δ*hilA* mutant. No significant differences were detected in the expression of *ipaJ* and *itrA* between the WT and Δ*hilA* strains (S6A and S6B Fig). Furthermore, there was no effect on the *hilA* mRNA level caused by the deletion of *itrA* in *Salmonella* (S6C Fig).

### Label-free quantitative phosphoproteomics demonstrates the massive impact of IpaJ on host protein phosphorylation

**Fig 5:**
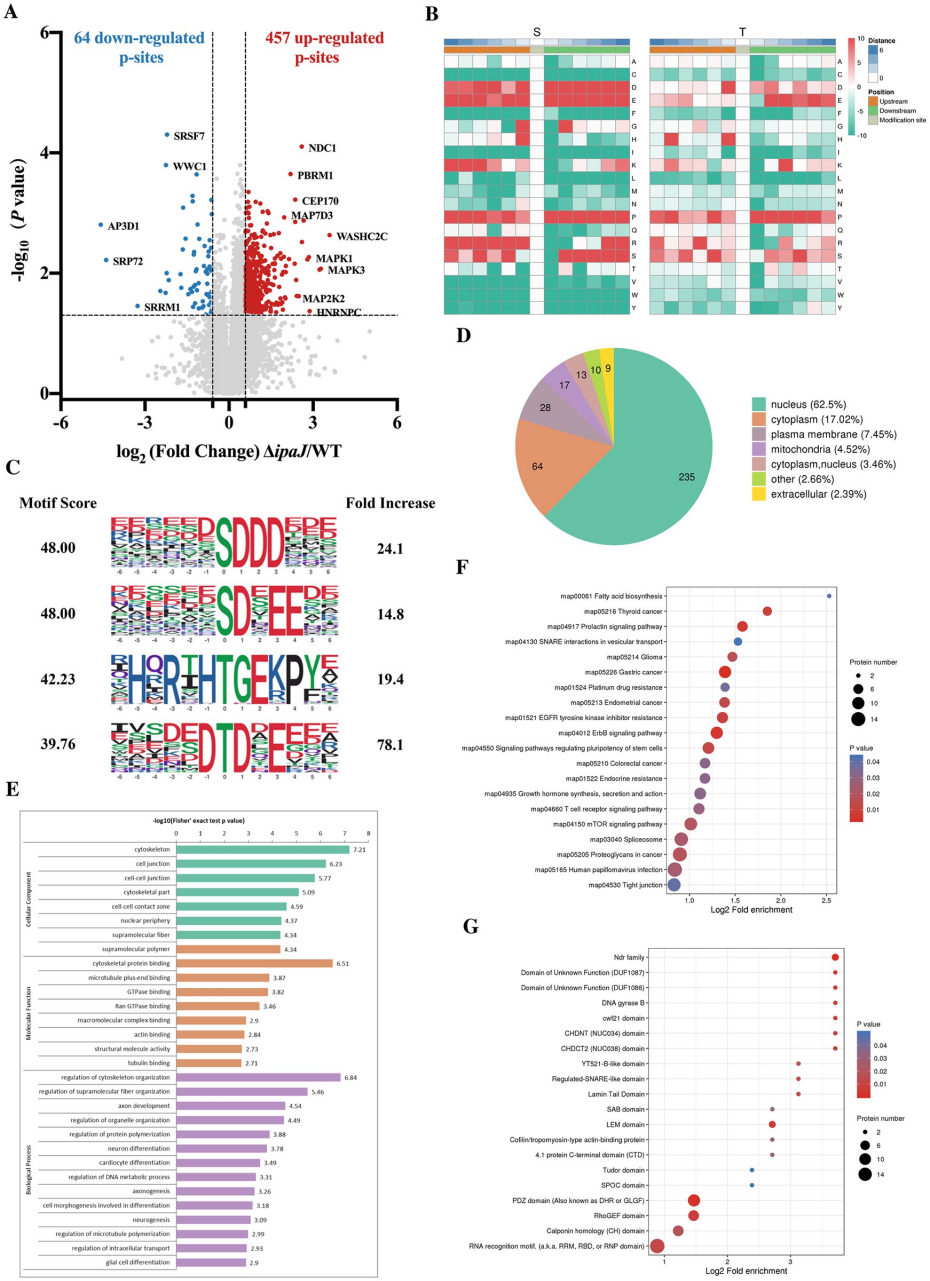
Phosphoproteomic profiles of proteins involved in dysregulated pathways. (**A**) Quantitative volcano map of differentially phosphosites between Δ*ipaJ* and WT infected groups. (**B**) Motif enrichment heat maps of amino acids upstream and downstream of all the identified phosphosites. (**C**) The predominant phosphorylation motif. (**D**) The subcellular localization of the differentially phosphorylated proteins between the two groups (Δ*ipaJ*/WT). (**E**) The distribution of the differentially phosphorylated proteins in GO secondary annotation between the two groups. (**F**) The enriched KEGG pathways related to the differentially phosphorylated proteins between the two groups. (**G**) The domain enrichment of differentially phosphorylated proteins between the two groups.

To assess the role of IpaJ as a virulence effector of T3SS1 in host cells and unravel the influence of IpaJ on host cell signaling pathways, quantitative phosphorylation proteomic analysis was performed, allowing the relative quantification of peptides by high-resolution MS/MS. Changes in protein phosphorylation levels were represented by theΔ*ipaJ*/WT (FC) group ratio, and a standard of localization probability > 0.75 was used to filter the identification data. A total of 20,259 phosphorylation sites on 4,875 proteins were identified, and 11,273 sites on 2,513 proteins contained quantitative information (S5 Table). Across three experimental replicates, we mapped 328 proteins and 457 phosphosites with a 1.5-fold increase and 60 proteins and 64 phosphosites with a 1.5-fold decrease (Fig 5A and S6 Table).

The frequency of amino acid residues flanking the phosphorylation sites was calculated in the mapped proteins to identify the phosphorylation motif. Among the protein sites with phosphorylated Ser, the proportion of Asp, Glu, and Pro was high, while that of Cys, Phe, Ile, Val, Trp, and Tyr was low (Fig 5B and Table A in S7 Table); the main motif was xxxxxx_S_DDDxxx (Fig 5C and Table B in S7 Table). Among the proteins with phosphorylated Thr, the proportion of Pro was high, while that of Phe and Leu was low; (Fig 5B), the main motif was xxxRxx_T_xExPxx (Fig 5C and Table B in S7 Table). Motif-based analysis extracted common MAPK and CDK (-S/T-P-), CAMK (-R-X-X-S/T-), and CK2 (-S/T-X-E-E-) consensus phosphorylation sequences (Table B in S7 Table). In addition to these kinase families, we also noted that kinases were phosphorylated in the assessment of phosphorylation regulation. These included MAPK (Mitogen-Activated Protein Kinase), CK1 (Casein Kinase 1), STK10 (Serine/Threonine Kinase 10), GSK3 (Glycogen Synthetase Kinase 3) and PAK2 (P21 (CDKN1A)-Activated Kinase 2), suggesting that these kinases may also play a role in protein phosphorylation by IpaJ during *S*. Pullorum infection, with MAPK being the most prominent regulator (Table B in S7 Table). Analysis of the subcellular localization of differentially phosphorylated proteins revealed that these differentially modified proteins were widely distributed in HeLa cells and were predominantly located in the nucleus (235/376, 62.5%), followed by the cytoplasm (64/376, 17.02%) and PM (28/376, 7.45%) (Fig 5D and Table B in S7 Table).

For an overall analysis of differentially phosphorylated proteins, Gene Ontology (GO), KEGG, and protein domain analyses of these proteins were performed. The results revealed that most of the proteins were related to the cytoskeleton, cytoskeletal protein binding, and the regulation of cytoskeletal organization (Fig 5E and S6 Table). These findings are consistent with the roles played by the cytoskeleton in host–pathogen interactions. KEGG analysis of differentially phosphorylated proteins revealed that the upregulated phosphorylated proteins were mainly involved in endocytosis, proteoglycans, spliceosomes, the mTOR signaling pathway, and tight junctions (Fig 5F and S6 Table), while the downregulated phosphorylated proteins were involved in the AMPK signaling pathway. Furthermore, the predominant phosphorylated domains in the phosphorylated proteins were the RNA recognition motif, the Postsynaptic density 95/discs large/zonula occludens-1 (PDZ) domain, and the guanine nucleotide exchange factor for Rholike GTPases (RhoGEF) domain (Fig 5G). In summary, these results indicate that IpaJ-altered phosphoproteins are involved in diverse biological functions, including the cell junction, cytoskeleton, and regulation of signaling pathways related to inflammatory responses.

### IpaJ inhibits the activation of the MAPK signaling pathway by downregulating p-MEK/p-ERK

**Fig 6:**
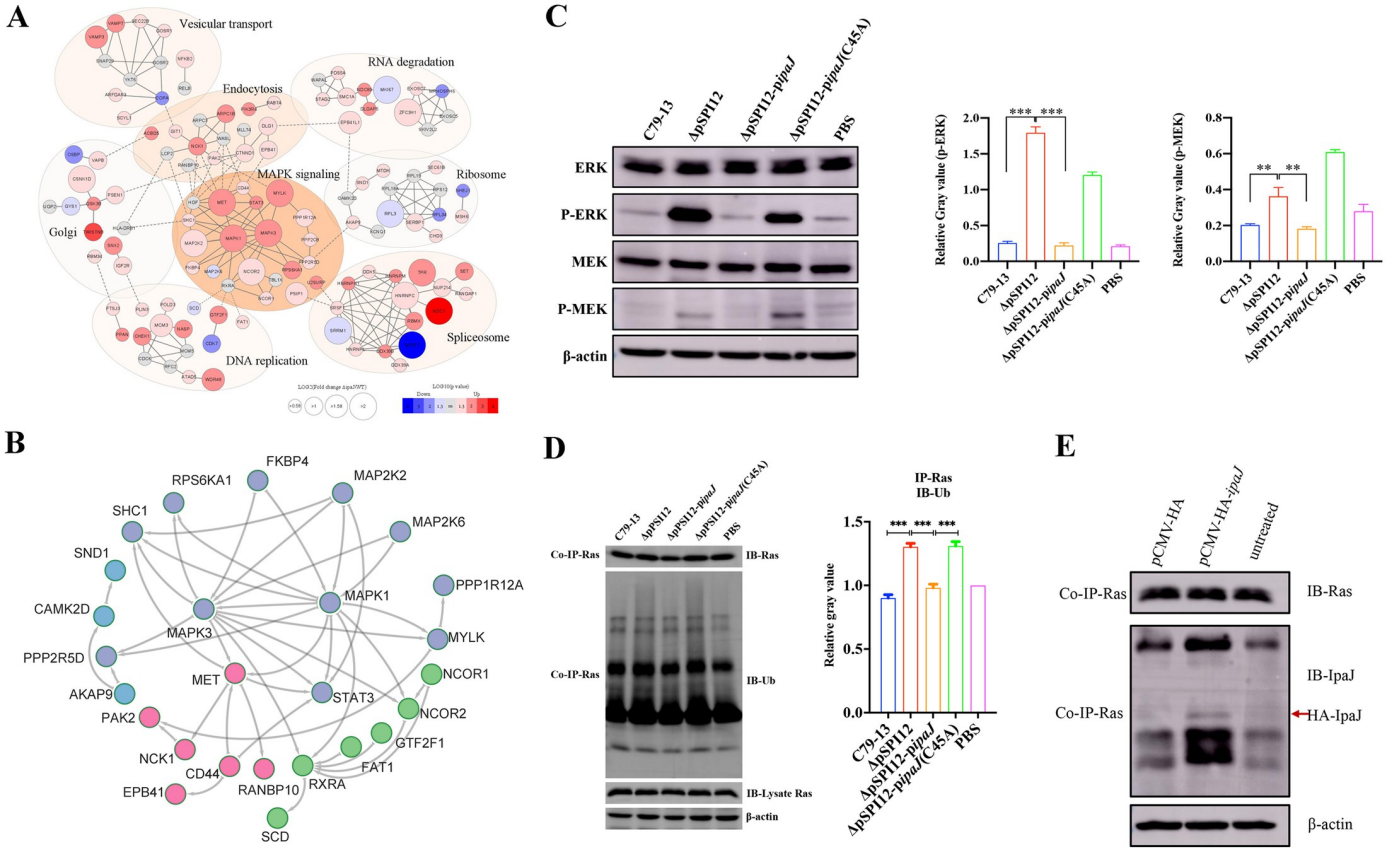
IpaJ inhibits the activation of the MAPK pathway (**A**) Graphical illustration of the differentially modified phosphoproteins using STRING (confidence score > 0.9). (**B**) Cluster analysis of K-means-based cytoskeleton-associated proteins. Regulated proteins were annotated, clustered, interacted and MAPK-associated cluster isolated. (**C**) Western blot analysis of signaling molecules (MEK and ERK) in the MAPK pathway in HeLa cells infected with WT or mutant strains at 6 h post-infection. (**D**) The HeLa cells infected with different *S*. Pullorum strains at 6 h post-infection were collected and subjected to IP using anti-Ras antibody. Ras expression levels and ubiquitination status were analyzed via Western blot using anti-Ras and antiubiquitin antibodies, respectively. (**E**) Interaction between IpaJ and Ras using Co-IP assay. The anti-Ras antibody was used to capture proteins from HeLa cells transfected with pCMV-HA-*ipaJ* or pCMV-HA plasmids. The captured proteins were subjected to Western blot analysis using the anti-IpaJ antibody. **: *P* < 0.01; ***: *P* < 0.001.

We next focused on defining the host signaling pathways affected by IpaJ-mediated alterations in the phosphoproteome of *Salmonella*-infected HeLa cells. A network representation of all differential expressed phosphoproteins was generated by STRING, displaying the top 50 proteins in interaction relationships (Fig 6A). Functional analysis revealed that these phosphorylated proteins in PPI were closely related to the cytoskeleton and spliceosome, consistent with GO and KEGG results.

Among the cluster of interacting proteins related to MAPK signaling pathway (Fig 6B), MAPK3 (T202 and Y204) and MAPK1 (T185 and Y187) phosphorylation sites showed the most significant multiples of difference, reaching folds of 9.4, 9.7, 6.9, and 7.2, respectively (S6 Table). MAPK1/3 activated by MEK phosphorylation plays a crucial role in cell growth, apoptosis, and immune responses. Among the cluster of interacting spliceosome proteins, HNRNPC (S138, 7.4) and SRSF7 (S192, 0.2) are known to be critical for mRNA splicing (Fig 6A).

To validate whether IpaJ inhibits the MAPK signaling pathways identified by label-free quantitative phosphoproteomic analysis, the signaling molecules ERK1/2, p-ERK1/2, MEK2, and p-MEK2 were selected for Western blot analysis in HeLa cells infected with the WT strain or its derivatives (ΔpSPI12, ΔpSPI12-P*ipaJ* and ΔpSPI12-P*ipaJ*C45A), respectively. As shown in Fig 6C, the expression levels of the two phosphorylated proteins p-ERK1/2 and p-MEK2 significantly decreased in HeLa cells infected with the IpaJ-expressed WT or ΔpSPI12-P*ipaJ* strain compared with those in HeLa cells infected with the ΔpSPI12 or ΔpSPI12-P*ipaJ*C45A strain, indicating that the representative proteins were dephosphorylated during *Salmonella* infection depending on the C39 protease activity of IpaJ. In addition, these results are consistent with those obtained from quantitative phosphoproteomic analysis. We further analyzed the upstream molecules Ras in the MAPK pathway and the results showed that ubiquitination of Ras was enhanced in the HeLa cells infected with the ΔpSPI12 or ΔpSPI12-P*ipaJ*C45A strain (Fig 6D). To identify the interaction between IpaJ and Ras, we transferred the eukaryotic expression plasmid pCMV-HA-*ipaJ* into HeLa cells and performed a Co-IP assay using an anti-Ras antibody (Beyotime, China), the HA-IpaJ was detected in the solution captured from cell lysates transferred with pCMV-HA-*ipaJ* (Fig 6E). These findings indicate that IpaJ can inhibit the degradation of Ras via the ubiquitin-proteasome pathway and subsequent MEK/ERK molecules, leading to the inhibition of the MAPK pathway. We then analyzed the cell viability during *Salmonella* infection using CCK8 assay. The cell viability was enhanced in HD-11 cells infected with ΔpSPI12 or ΔpSPI12-p*ipaJ*(C45A) strains than in macrophages infected with WT or complementary strains (S7 Fig).

## Discussion

As a highly expressed protein during *Salmonella* infection, IpaJ was first identified to be an antigen specific to *S*. Pullorum by suppression subtractive hybridization (SSH) [14]. Thereafter, IpaJ was demonstrated to be responsible for the inhibition of the NF-κB signaling pathway and the subsequent inflammatory response [17]. However, whether IpaJ acts as an effector of *Salmonella* T3SS was unknown. Our Tn-Seq screening results revealed that *hilC* and *hilA* played a role in modulating the expression of IpaJ. We further found that the secretion of IpaJ into bacterial culture supernatants is dependent on HilA. The SPI-1 regulators HilD, HilA, and InvF positively control the expression of genes within the SPI-1 island in a cascade manner, wherein HilD induces the expression of HilA, which in turn activates *invF* and SPI-1 genes that encode components and effectors of the SPI-1/T3SS apparatus [18]. Therefore, the fact that the secretion of IpaJ was not detected in either *hilA-* or SPI-1-deleted strains indicates that IpaJ is an effector of the SPI-1/T3SS apparatus. In contrast, *ssrB* (of the SPI-2 two-component regulatory system)- and SPI-2-deleted strains showed identical levels of IpaJ expression and secretion, indicating that IpaJ is not an effector of the SPI-2/T3SS apparatus. Moreover, the SPI-19-deleted *Salmonella* strain showed significantly decreased IpaJ secretion because of the close relationship between T3SS1 and SPI-19 [19]. Our previous study revealed that deletion of SPI-19 decreased the expression of SPI-1 genes (*hilA*, *hilD*) in *S*. Pullorum and the subsequent bacterial invasion defect in host cells [20]. Therefore, the IpaJ displayed weaker secretion in ΔSPI-19 than in WT strain. SPI-1/T3SS plays a role in *Salmonella* invasion into host cells. The deletion of *ipaJ* in *S*. Pullorum also decreased bacterial entry into chicken cells [15]. Although SPI-1 regulators can control the secretion of IpaJ, they cannot bind to the promoter of *ipaJ* to regulate its transcription. We speculated that the concentration of chloramphenicol used (250 μg/ml) was relatively high for screening out the direct regulators controlling *ipaJ* expression *in vivo*. Therefore, it is essential to identify the direct regulators of *ipaJ in vitro* using DNA pull-down assay.

Using a DNA pull-down assay and EMSA, we identified ItrA, a transcription regulator of the DeoR family, as the direct regulator controlling the transcription of *ipaJ*. DeoR family regulators are widely distributed in bacteria, and act as transcriptional repressors or activators in multiple physiological processes, including sugar and nucleotide metabolism, virulence, and secondary metabolism [21,22]. These regulators, composed of approximately 250 amino acids, contain a conserved N-terminal region that includes a DNA-binding motif. The C-terminal region is speculated to be responsible for oligomerization into dimers, tetramers, and hexamers [23–25]. Most DeoR family regulators serve as repressors, such as DeoR from *E*. *coli*, and FruR from *Streptococcus gordonii* [26,27], whereas some regulators can promote virulence gene expressions, such as IgeR from *S*. Typhi and SetA from *Pseudomonas syringae* [22,25]. Here we found that ItrA composed of 255 amino acids, acts as a positive regulator controlling the transcription of *ipaJ* in *Salmonella*.

IpaJ, a cysteine protease effector of *Shigella*, can cleave the peptide bond between N-myristoylated Gly2 and Asn3 in proteins [13]. Myristoylome profiling has revealed that IpaJ can efficiently cleave most N-myristoylated proteins, which are involved in cellular growth, signal transduction, autophagosome maturation, and organelle function, *in vitro* [28]. IpaJ also exhibits strong specificity towards Golgi-associated ARF/ARL GTPases and the E3 ubiquitin ligase ZNRF2 *in vivo* [29]. Both IpaJ proteins share almost 49% homology and contain conserved C, H, and D amino acids. Therefore, they have the same functions in inhibiting the inflammatory response and disrupting the Golgi apparatus [17]. In the present study, phosphoproteomic analysis revealed that IpaJ is likely to play a crucial role in the MAPK signaling pathways, involved in cell proliferation, apoptosis, inflammation, etc. At the early stage of *Salmonella* infection, one evident manifestation is actin cytoskeleton rearrangement [30]. Our phosphoproteomic analysis revealed that the actin cytoskeleton is central to IpaJ-mediated cell-autonomous immunity during bacterial infections. The predominant target proteins regulated by IpaJ belong to various signaling pathways, including MAPK, WASH, Rho GTPase, P53, and MET/mTOR signaling pathways. Western blot analysis confirmed that the p-MEK and p-ERK molecules were significantly decreased by IpaJ, indicating that IpaJ inhibits the activation of the MAPK pathway at the early stage of *Salmonella* infection to promote immune evasion.

**Fig 7:**
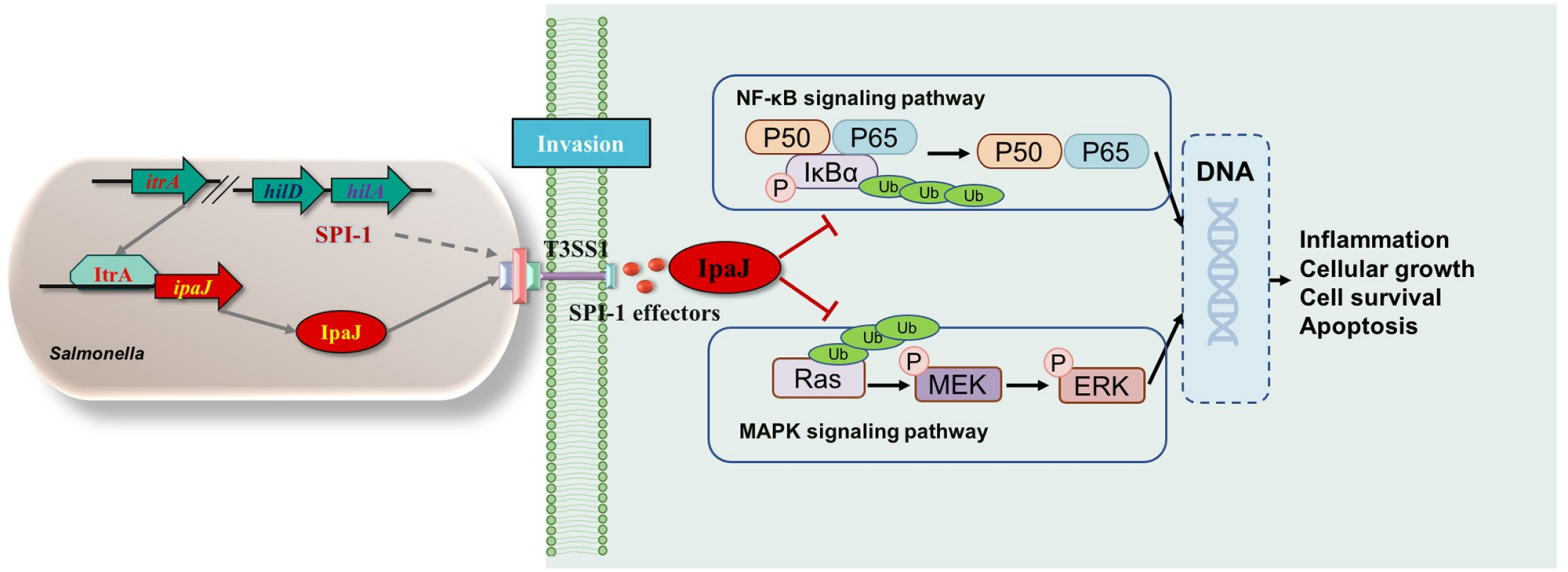
Schematic illustration of IpaJ as a T3SS1 effector protein that can suppress cellular immune responses during *Salmonella* infection. The DeoR family regulator ItrA directly binds to the promoter of *ipaJ* and initiates its expression. The expressed IpaJ is then secreted into host cells or bacterial culture supernatants depending on SPI-1/T3SS1, which is regulated by HilA and HilD. As an effector protein of SPI-1, IpaJ can prevent the ubiquitination and degradation of IκBα in the NF-κB signaling pathway and inhibit the phosphorylation of MEK and ERK in the MAPK signaling pathway through deubiquination of Ras, thereby downregulating proinflammatory responses, cellular growth and differentiation, cell survival, and apoptosis.

Collectively, we propose a model for the expression, secretion, and function of IpaJ: At the early stage of *Salmonella* infection, the DeoR family regulator ItrA directly binds to the promoter of *ipaJ* to initiate the expression of IpaJ. The expressed IpaJ is then secreted into host cells in a T3SS1-dependent manner. In the host cells, IpaJ can downregulate the expression level of p-MEK/p-ERK molecules to inhibit the activation of the MAPK pathways and the subsequent proinflammatory responses, promoting immune evasion and facilitating bacterial survival in the host (Fig 7). However, the enzyme activity and substrates of IpaJ needs to be revealed in the further study.

## Materials and methods

### Bacterial strains and plasmids

The bacterial strains used in the present study are listed in S1 Table. The bacteria were cultured under static conditions in Luria–Bertani (LB) broth at 37°C. The *E*. *coli* χ7213 λpir strain was used as the conjugation donor to introduce suicide plasmids into *S*. Pullorum strains. The deletion strains used in the Tn-Seq and DNA pull-down assays were constructed using the λ-red recombination system; all the primers used are listed in S2 Table. When required, the following antibiotics were added: carbenicillin (Carb, 100 μg/ml), chloramphenicol (Cm, 32 μg/ml), and kanamycin (Km, 100 μg/ml).

### Genetic engineering of *S*. Pullorum

In-frame deletion mutants were constructed using *sacB*-based allelic exchange vectors, as described previously [15]. In brief, the Gibson assembly method was used to ligate the PCR-amplified upstream and downstream fragments into the suicide vector pDM4. The recombinant plasmids were then transformed into *E*. *coli* χ7213 *λpir* and subsequently transferred into *S*. Pullorum C79-13 through conjugation. Finally, the mutants were selected and cultured on LB medium containing 20% (w/v) of L-sucrose [15]. Reporter strains with a promoterless Cm resistance gene (*cm*^*R*^) inserted downstream of the *ipaJ* start codon (CCACGTATG) were constructed using the same steps employed for the generation of in-frame mutants. All the primers used to construct and validate the C79-13-P*ipaJ*-*cm* strains are listed in S2 Table.

### Transposon insertion sequencing (Tn-Seq)

Modified pSC189 carrying a tetracycline resistance gene was used to construct the transposon library [18]. The library was resuspended in 5 ml LB medium with (output) or without (input) Cm and cultured at 37°C for 24 h. Then, the cultures in each group were plated on LB medium and incubated at 37°C for 12 h. Finally, all the colonies in each group were collected from the plates and subjected to the genomic extraction and sequencing, as described previously [31]. The library was sequenced on the HiSeq 2500 platform (Illumina, San Diego, CA) by GENEWIZ (Suzhou, China). Reads for each output library were normalized on the basis of the input library, and the reads per TA site were tallied and assigned to annotated genes or intergenic regions, as described [31]. The fold change (FC) and Mann–Whitney U test (MWU) of each locus are based on comparisons of the output and input libraries.

### Secretion proteins assay and immunoblot analysis

The bacterial strains were inoculated in LB medium at 37°C for 24 h. Each culture of different *S*. Pullorum strains was centrifuged, the supernatant was passed through a 0.22-μm filer (Merck Millipore, Germany), and trichloroacetic acid at a final concentration of 10% (v/v) was added to the supernatant. The treated supernatant samples were incubated at 4°C overnight. The precipitated proteins were centrifuged at 14,000 ×g for 30 min at 4°C and then washed in ice-cold acetone. Following this, the precipitated proteins were washed four times and then boiled in 5× sample buffer.

Bacterial cell pellets or concentrated secretion proteins were suspended in PBS to normalize the culture densities based on BCA Protein Assay Kit (Beyotime, China) measurements for immunoblot analyses. In total, 20 μl of each normalized sample was loaded onto 12% denaturing polyacrylamide gels. The proteins were resolved by electrophoresis and then transferred to PVDF membranes (PALL, USA). The membranes were incubated in 5% bovine serum albumin (BSA) blocking buffer; analyzed by immunoblotting using mouse anti-IpaJ monoclonal antibody (1:5000, Laboratory stock), mouse anti-HilA polyclonal antibody (1:2000, laboratory stock), and mouse anti-DnaK monoclonal antibody (1:5000, ab69617, Abcam, UK) as controls; and finally incubated with the secondary antibody Goat Anti-Mouse IgG (H&L)-HRP (ab6708, Abcam, UK) at a 1:10000 dilution. ECL reagent (Thermo Fisher, USA) was used to visualize the blots.

### CCF2-AM FRET assay

IpaJ-TEM-1 fusions were constructed by cloning *ipaJ* into the TEM-1 fusion cloning vector, pCX340. The recombinant plasmid pCX340-*ipaJ* was then introduced into the WT, ΔSPI-1, and ΔSPI-2 strains, which were then used to infect HeLa cells. Cells infected with the WT-pCX340 strain was used as the negative control. After infection (3 h in DMEM supplemented with 1mM IPTG), cells were washed and loaded with CCF2-AM (Thermo Fisher, USA). Blue and green fluorescence were monitored using a Leica TCS SP8 STED inverted fluorescence microscope (Leica, Germany).

### DNA pull-down assay

Overnight cultures of C79-13 were subcultured in LB medium at 37°C for 4 h. Bacterial pellets were collected, washed using ddH_2_O, and stored at −80°C. The pellets were resuspended in 20 ml lytic fluid buffer (20% of sucrose, 4% of CHAPS, and 40 mmol/l DTT) under 700 Mpa high-pressure crushing for 3 min. The bacteria were cracked by ultrasonication, and the cracking supernatant was collected after centrifugation. Biotinylated DNA and NeutrAvidin Agarose Resin beads (ThermoFisher, USA) were mixed and incubated at 25°C for 1 h. Following this, the probe-labeled beads were washed twice with TE and BS/THES buffers. The probe-labeled beads and supernatant lysates were then mixed and incubated with slow shaking at 25°C for 30 min. After washing four times with BS/THES buffer, the binding proteins were eluted from the beads with a NaCl concentration gradient. Finally, the beads were eluted with ddH_2_O to release biotin-DNA for SDS-PAGE analysis. The experiments were performed three times and confirmed to be stable with similar bands by SDS-PAGE analysis. Finally, the proteins eluted with 500 nM and 750 nM NaCl buffer were submitted to GeneCreate (Wuhan, China) for mass spectrometry (MS).

### Electrophoretic mobility shift assay (EMSA)

In brief, *itrA* was amplified from C79-13 genomic DNA and cloned into the plasmid pColdI, which was then transformed into *E*. *coli* BL21 (DE3) to express the rHis-ItrA protein. The expression protein was purified using the Ni-NTA Purification Kit (ThermoFisher, USA) according to the manufacturer’s instructions and stored at −80°C for EMSA.

EMSA was performed as follows: FAM-labeled PCR fragments encompassing the regulatory regions of *ipaJ* were amplified from C79-13 using the primer pairs P*ipaJ*-EMSA-F/R (S2 Table). Each PCR product (25 ng) was mixed with increasing concentrations of purified rHis-ItrA in a buffer containing 10 mM Tris-HCl (pH 8.0), 50 mM KCl, 1 mM DTT, 0.5 mM EDTA, 5% glycerol, 10 μg/ml BSA, and 200 ng/μl Poly(dI:dC). The reaction mixtures were incubated at 37°C for 30 min, and the protein–DNA complex was then separated by 6% native PAGE on ice. The DNA fragments were stained with ethidium bromide and displayed by Fluorescence image scans (Typhoon FLA9500, GE, USA).

### Immunofluorescence microscopic analysis of ASC specks

HD-11 cells were seeded on coverslips at a density of 1.5 × 10^5^ cells per well in 24-well plates and cultured overnight. After stimulation as indicated, the cells were washed with PBS, fixed with 4% paraformaldehyde for 15 min, permeabilized with 0.1% Triton X-100 for 5 min, and blocked with 5% FBS in TBSTx (10mM Tris-HCl,150mM NaCl, 20% Tween, and 0.1% Triton X-100) for 60 min. The cells were then incubated with anti-IpaJ antibody 4G6 (1:100) [32], followed by anti-mouse IgG (ab150113, Abcam, UK) and DAPI (D3571, Invitrogen, USA). The pYA3334-RFP plasmid was transformed into WT, ΔSPI-1, and ΔSPI-2 strains to express the red fluorescence protein. Images were acquired using a Leica TCS SP8 STED inverted fluorescence microscope (Leica, Germany).

### Protein extraction and trypsin digestion

Proteomic sequencing samples in lysis buffer (8 M urea and 1% protease inhibitor cocktail) were sonicated thrice on ice using a high-intensity ultrasonic processor (Scientz, China). Following this, the remaining debris was removed after centrifugation at 12,000 ×g at 4°C for 10 min. Finally, the protein concentration in the supernatant was determined using the BCA Protein Assay Kit (Beyotime, China), according to the manufacturer’s instructions. For digestion, the protein solution was treated with 5 mM dithiothreitol for 30 min at 56°C and alkylated with 11 mM iodoacetamide for 15 min at room temperature in darkness. The protein sample was then diluted by adding 100 mM TEAB with a urea concentration less than 2M. Finally, trypsin was added at a 1:50 trypsin-to-protein mass ratio for the first overnight digestion overnight and at a 1:100 trypsin-to-protein mass ratio for the second 4-h digestion.

### TMT/iTRAQ labeling and LC-MS/MS analysis

After trypsin digestion, the peptide was desalted using a Strata X C18 SPE column (Phenomenex, USA) and vacuum-dried. Following this, the peptide was reconstituted in 0.5 M TEAB and processed according to the manufacturer’s protocol of the TMT kit/iTRAQ kit. In brief, one TMT/iTRAQ reagent unit was thawed and reconstituted in acetonitrile.

The tryptic peptides were dissolved in 0.1% formic acid (solvent A) and directly loaded onto a homemade reversed-phase analytical column (15 cm length, 75 μm i.d.). The gradient consisted of an increase from 6% to 23% of solvent B (0.1% formic acid in 98% acetonitrile) over 26 min, an increase from 23% to 35% in 8 min, and an increase to 80% in 3 min, followed by holding at 80% for the last 3 min, all at a constant flow rate of 400 nl/min on an EASY-nLC 1000 UPLC system. The peptides were subjected to an NSI source, followed by tandem mass spectrometry (MS/MS) coupled online to UPLC. The applied electrospray voltage was 2.0 kV. The m/z scan range was 350–1800 m/z for a full scan, and intact peptides were detected using Orbitrap at a resolution of 70,000. Peptides were then selected for MS/MS with the NCE setting being 28, and fragments were detected using Orbitrap at a resolution of 17,500. The data-dependent procedure alternated between one MS scan and 20 MS/MS scans with 15.0 s dynamic exclusion. Automatic gain control (AGC) was set at 5E4. The fixed first mass was set at 100 m/z.

### Database search

The resulting MS/MS data were processed using the MaxQuant search engine (v.1.5.2.8). Tandem mass spectra were searched against the human Uniprot database concatenated with a reverse decoy database. Trypsin/P was specified as the cleavage enzyme allowing up to four missing cleavages. The mass tolerance for precursor ions was set at 20 ppm in the first search and 5 ppm in the main search, and the mass tolerance for fragment ions was set at 0.02 Da. Carbamidomethylation on Cys was specified as a fixed modification. Acetylation and oxidation on Met were specified as variable modifications. FDR was adjusted to less than 1%, and the minimum score for modified peptides was set to more than 40.

### Co-immunoprecipitation (Co-IP)

HeLa cells cultured in 6-well plates were infected with bacteria (MOI = 100:1) at 5% CO_2_ and 37°C. After 3 h of infection, the medium was replaced with fresh DMEM supplemented with 100 mg/ml gentamicin and 25 ng/ml TNF-α and incubated for 30 min. The cells were washed three times with cold PBS and collected for immunoprecipitation. HeLa cells were seeded into 24-well plates and transfected with pCMV-HA-*ipaJ* or pCMV-HA the next day using the Lipofectamine 3000 kit (Invitrogen, CA, USA) according to the manufacturer’s protocol. At 24 h post-transfection, the cells were treated with epidermal growth factor (EGF) (25 ng/ml, Absin) for 5 min and cell pellets were collected for immunoprecipitation.

Cell pellets were resuspended in lysis buffer containing a protease inhibitor cocktail (20mM Tris-HCl, 150mM NaCl and pH 7.5, 0.2%NP-40, 1×protease inhibitor cocktail). The mixture was then centrifuged at 14,000 × g for 5 min. The resulting supernatant was incubated overnight at 4°C (with rotation) with purified protein A+G magnetic bead-conjugated anti-Ras antibodies, as appropriate. The beads were washed three times with lysis buffer and analyzed by Western blot with anti-IpaJ antibody (1:5000, 4G6) or anti-ubiquitin antibody (1:2000, ab19247, Abcam).

### Statistical analysis

All data are expressed as the mean ± standard error of the mean (SEM) values, unless specified otherwise. Data were analyzed using GraphPad Prism 8.0 (La Jolla, CA, USA) to detect the differences among the treated groups (La Jolla, CA, USA). *P*-values < 0.05 were considered statistically significant when using one-way analysis of variance (ANOVA).

## Supporting information

S1 Fig Expression of IpaJ in the C79-13 strain cultured in LB. The expression of IpaJ was assessed by Western blot analysis using monoclonal anti-IpaJ antibody (4G6) and polyclonal anti-HilA antibodies. Whole cell lysates were prepared from bacterial cultures grown in LB medium at 37°C. The samples were collected every 2 h at the indicated OD600 values. The expression of DnaK was determined using a monoclonal anti-DnaK antibody as the control.
(TIF)

S2 Fig IpaJ secretion depends on SPI-1/T3SS1. The *ipaJ* ORF was inserted into plasmid pBAD33 with an arabinose-inducible promoter. pBAD33-*ipaJ* was transformed into Δ*hilA* and ΔSPI-1 mutants. Arabinose induced the expression of IpaJ in Δ*hilA-*pBAD33*-ipaJ* and ΔSPI-1-pBAD33-*ipaJ* strains. The expressed IpaJ was not able to be secreted into the supernatant without SPI-1/T3SS1. DnaK was used as the control.
(TIF)

S3 Fig Secretion of IpaJ into HeLa cells depends on SPI-1/T3SS1 using the CCF2-AM FRET assay. The WT, ΔSPI-1, and ΔSPI-2 strains carrying pCX340*-ipaJ* were used to infect HeLa cells. At 3h post-infection, the fluorescence in the cells was evaluated using confocal microscopy. Blue fluorescence indicates that IpaJ is translocated into the cells, whereas green fluorescence does not.
(TIF)

S4 Fig DNA-Pull down by the *ipaJ* promoter region (P*ipaJ*). The DNA fragments containing P*ipaJ* or *ipaJ* open reading frame (*ipaJ* orf) were labeled with biotin and fixed to agarose beads. The probe-labeled beads were then mixed with excess poly (dI:dC) and lysates of the C79-13 strain, washed, eluted with a concentration gradient of NaCl and ultimately treated with ddH2O at 70°C to release bound proteins, followed by silver staining analysis. The arrows indicate different eluted proteins between the two groups.
(TIF)

S5 Fig Binding of ItrA to P*ipaJ* using different concentrations of rHis-ItrA. FAM-labeled probes from P*ipaJ* were used for EMSA with 0, 0.1, 1, 2, 4 or 8 μM of purified rHis-ItrA.
(TIF)

S6 Fig qRT-PCR analysis of *hilA*, *itrA*, and *ipaJ* expression of in *Salmonella*. (**A**) mRNA levels of *itrA* in WT and Δ*hilA* strains. The Δ*itrA* mutant was used as a negative control. (**B**) mRNA levels of *ipaJ* in WT and Δ*hilA* strains. (**C**) mRNA levels of *hilA* in WT and Δ*itrA* strains. The Δ*hilA* mutant was used as a negative control.
(TIFF)

S7 Fig Proliferation of HD-11 cells infected with *Salmonella*. Cell viability was determined using CCK8 assay. The number of cells infected with the WT strain was set at 100%. *: *p* < 0.05.
(TIFF)

S1 Table *Salmonella* strains and plasmids used in this study. (DOCX)

S2 Table Primers used in this study. (DOCX)

S3 Table Putative activators of *ipaJ* identified by Tn-Seq. (DOCX)

S4 Table MS results of proteins screened by DNA pull-down assay. (DOCX)

S5 Table Results of label-free quantitative phosphoproteomics. (XLSX)

S6 Table The differentially expressed phosphorylated proteins between two groups. (XLSX)

S7 Table The motifs of phosphorylated proteins. **(A**) Motif analysis of phosphorylated proteins; (**B**) Annotation of phosphorylated motifs.
(XLSX)
