# Supplementary figures and images for: The Salmonella T3SS1 effector IpaJ is regulated by ItrA and inhibits the MAPK signaling pathway
Source: PLoS Pathog. 2022 Dec 7;18(12):e1011005. doi: 10.1371/journal.ppat.1011005 (PMC9728880; doi:10.1371/journal.ppat.1011005)

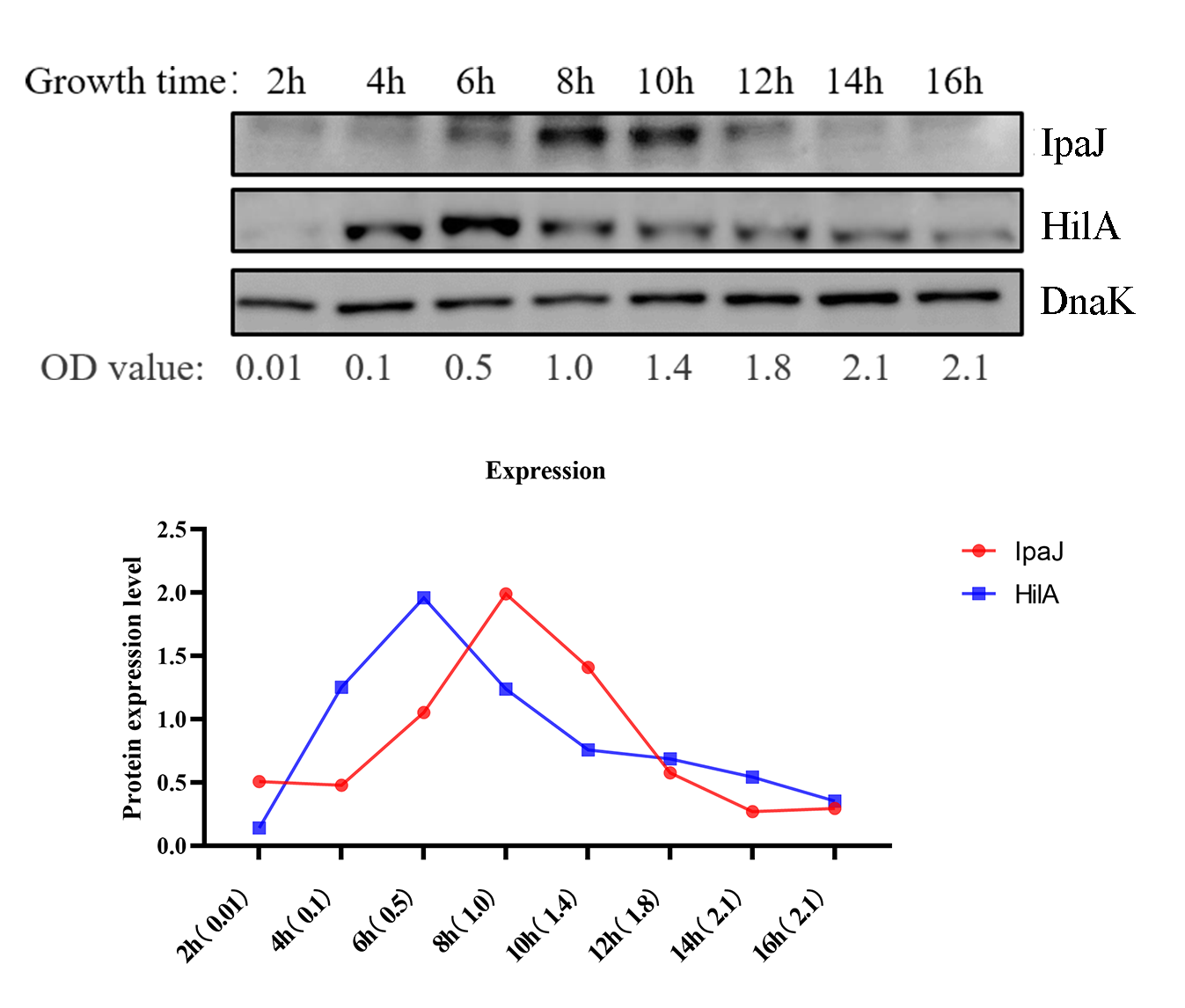

Supplement: S1 Fig — The expression of IpaJ was assessed by Western blot analysis using monoclonal anti-IpaJ antibody (4G6) and polyclonal anti-HilA antibodies. Whole cell lysates were prepared from bacterial cultures grown in LB medium at 37°C. The samples were collected every 2 h at the indicated OD600 values. The expression of DnaK was determined using a monoclonal anti-DnaK antibody as the control. (TIF) [file ppat.1011005.s001.tif]

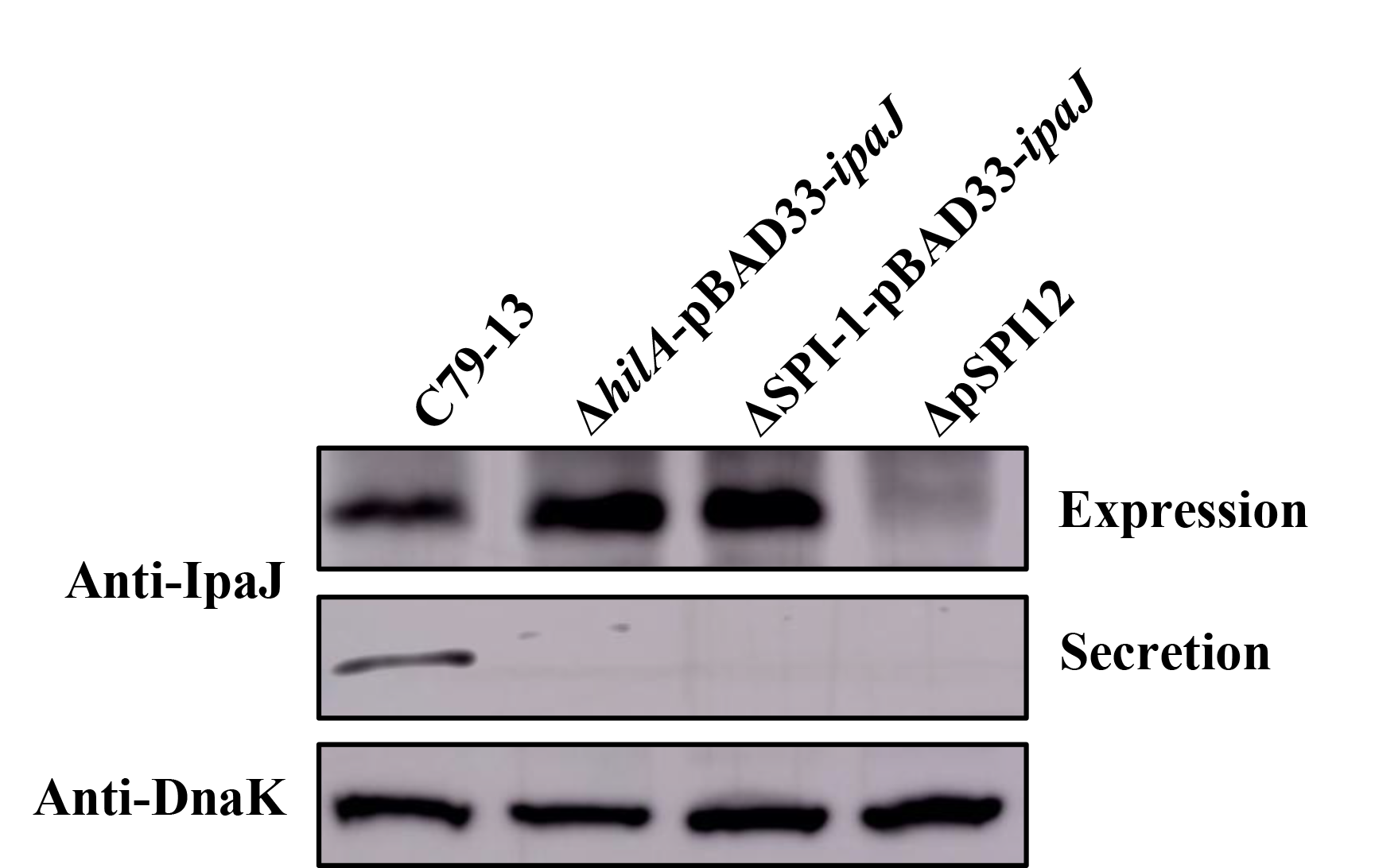

Supplement: S2 Fig — The ipaJ ORF was inserted into plasmid pBAD33 with an arabinose-inducible promoter. pBAD33-ipaJ was transformed into ΔhilA and ΔSPI-1 mutants. Arabinose induced the expression of IpaJ in ΔhilA-pBAD33-ipaJ and ΔSPI-1-pBAD33-ipaJ strains. The expressed IpaJ was not able to be secreted into the supernatant without SPI-1/T3SS1. DnaK was used as the control. (TIF) [file ppat.1011005.s002.tif]

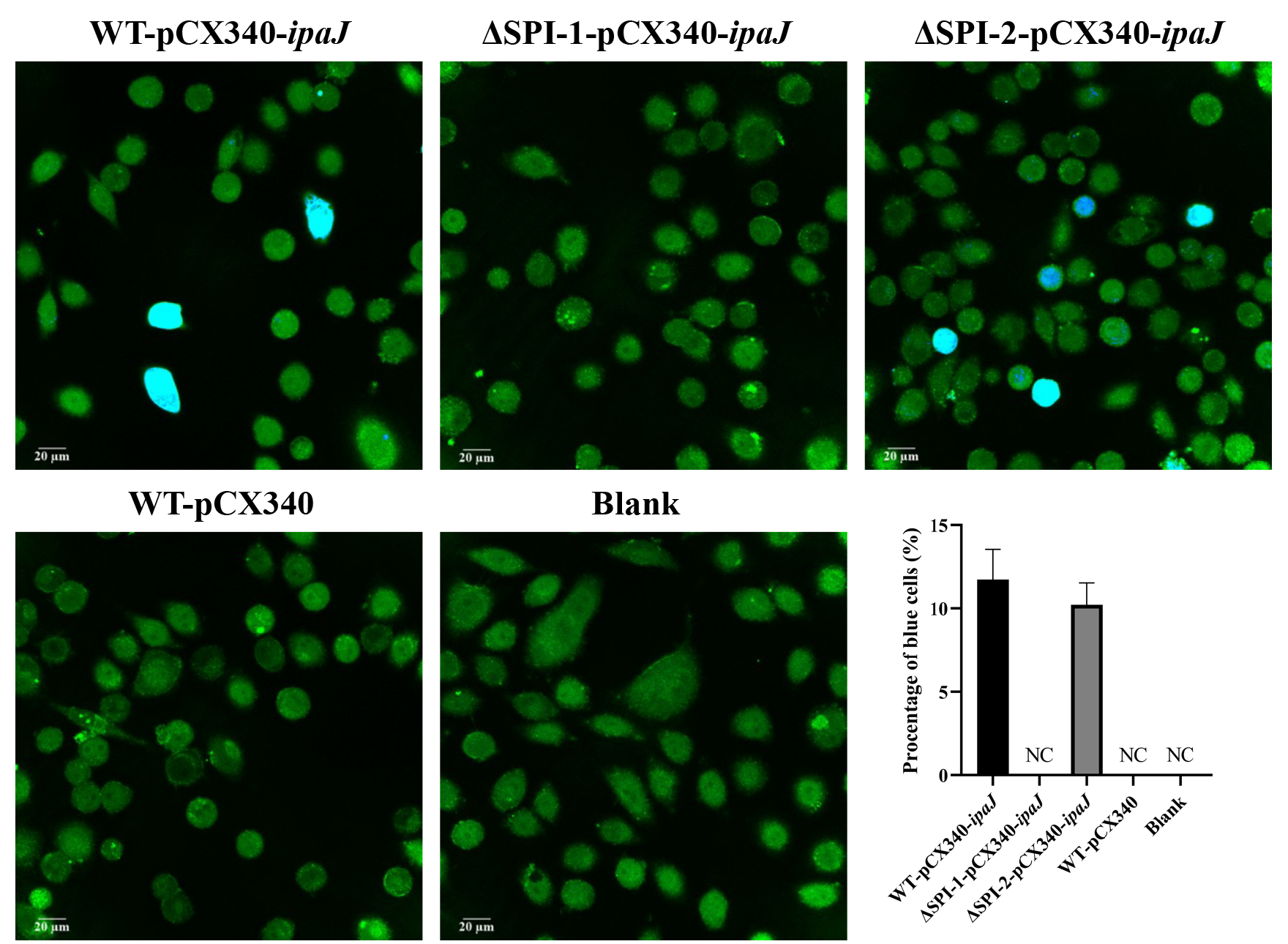

Supplement: S3 Fig — The WT, ΔSPI-1, and ΔSPI-2 strains carrying pCX340-ipaJ were used to infect HeLa cells. At 3h post-infection, the fluorescence in the cells was evaluated using confocal microscopy. Blue fluorescence indicates that IpaJ is translocated into the cells, whereas green fluorescence does not. (TIF) [file ppat.1011005.s003.tif]

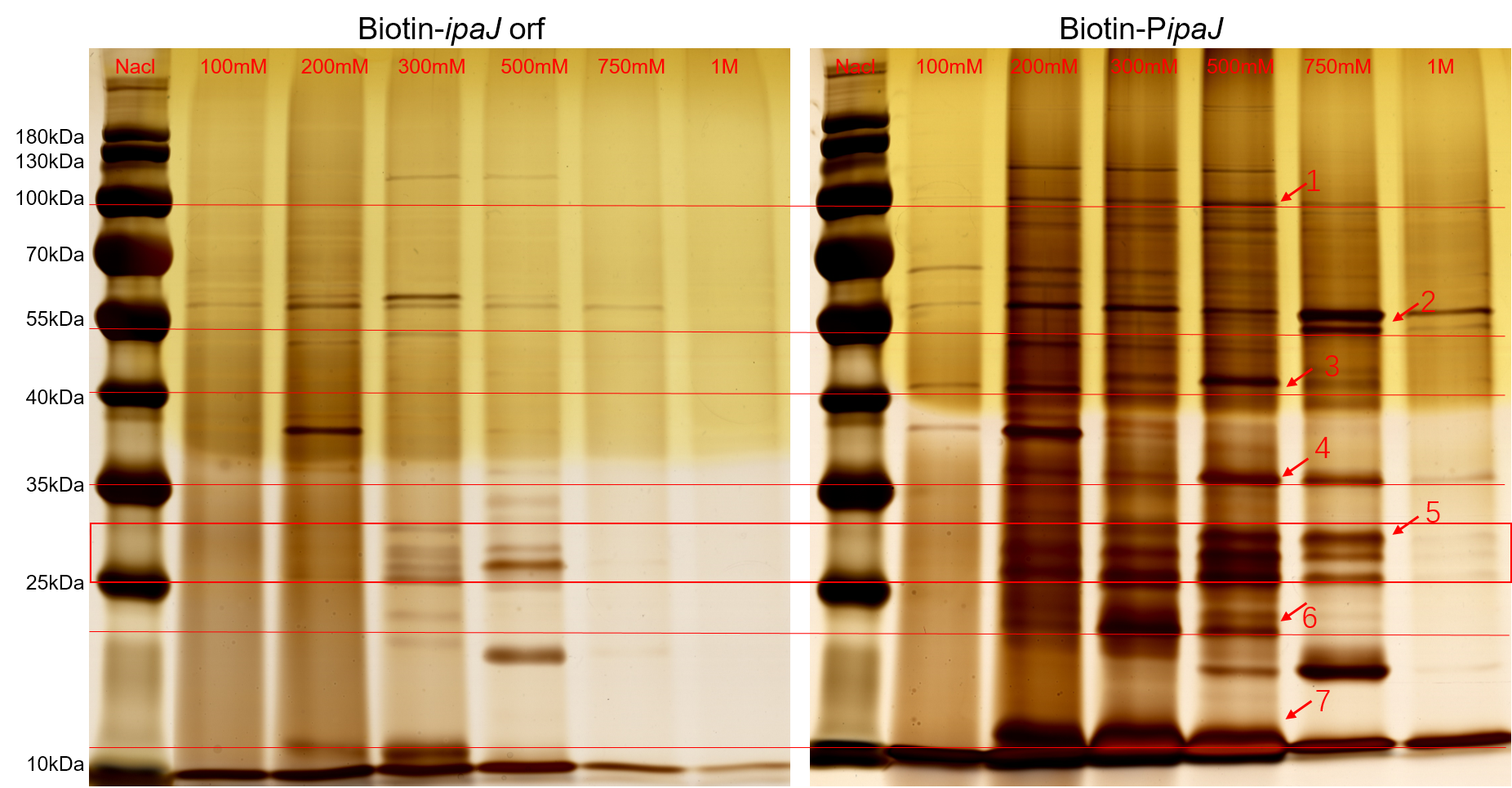

Supplement: S4 Fig — The DNA fragments containing PipaJ or ipaJ open reading frame (ipaJ orf) were labeled with biotin and fixed to agarose beads. The probe-labeled beads were then mixed with excess poly (dI:dC) and lysates of the C79-13 strain, washed, eluted with a concentration gradient of NaCl and ultimately treated with ddH2O at 70°C to release bound proteins, followed by silver staining analysis. The arrows indicate different eluted proteins between the two groups. (TIF) [file ppat.1011005.s004.tif]

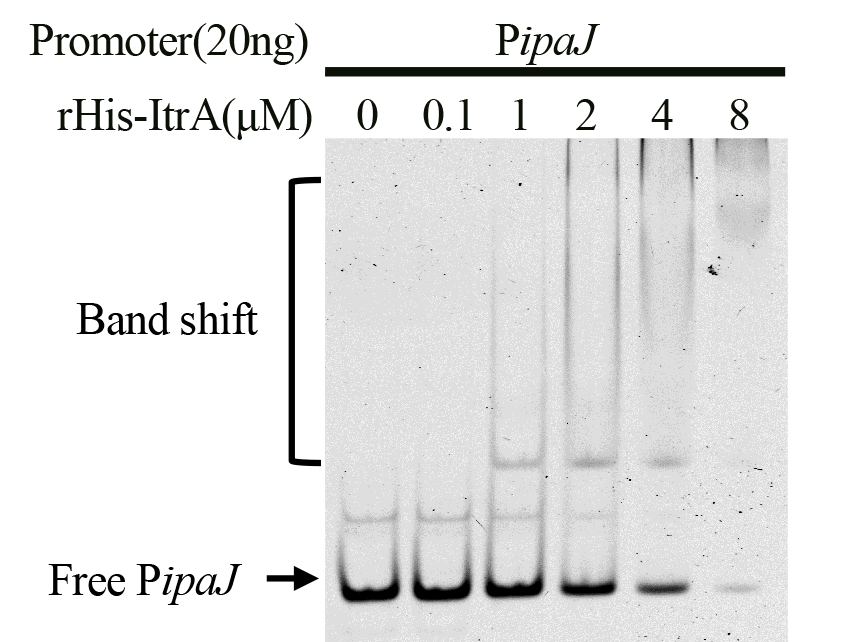

Supplement: S5 Fig — FAM-labeled probes from PipaJ were used for EMSA with 0, 0.1, 1, 2, 4 or 8 μM of purified rHis-ItrA. (TIF) [file ppat.1011005.s005.tif]

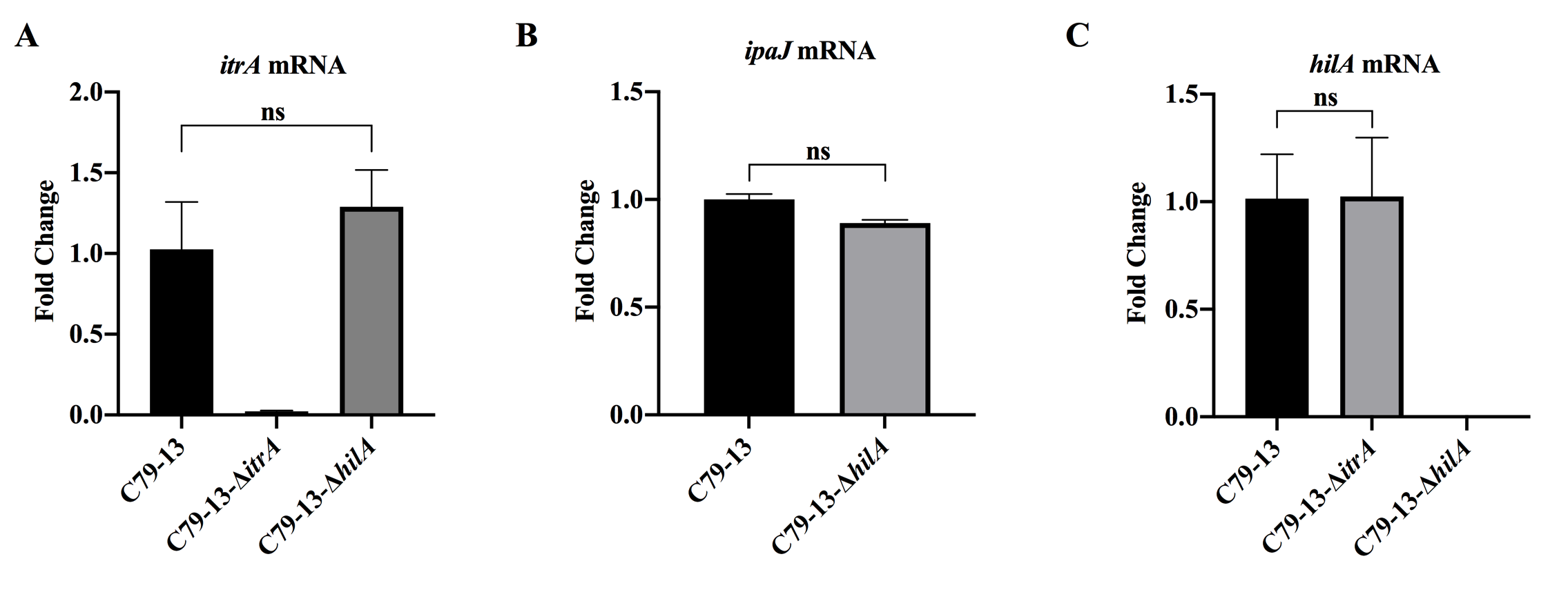

Supplement: S6 Fig — (A) mRNA levels of itrA in WT and ΔhilA strains. The ΔitrA mutant was used as a negative control. (B) mRNA levels of ipaJ in WT and ΔhilA strains. (C) mRNA levels of hilA in WT and ΔitrA strains. The ΔhilA mutant was used as a negative control. (TIFF) [file ppat.1011005.s006.tiff]

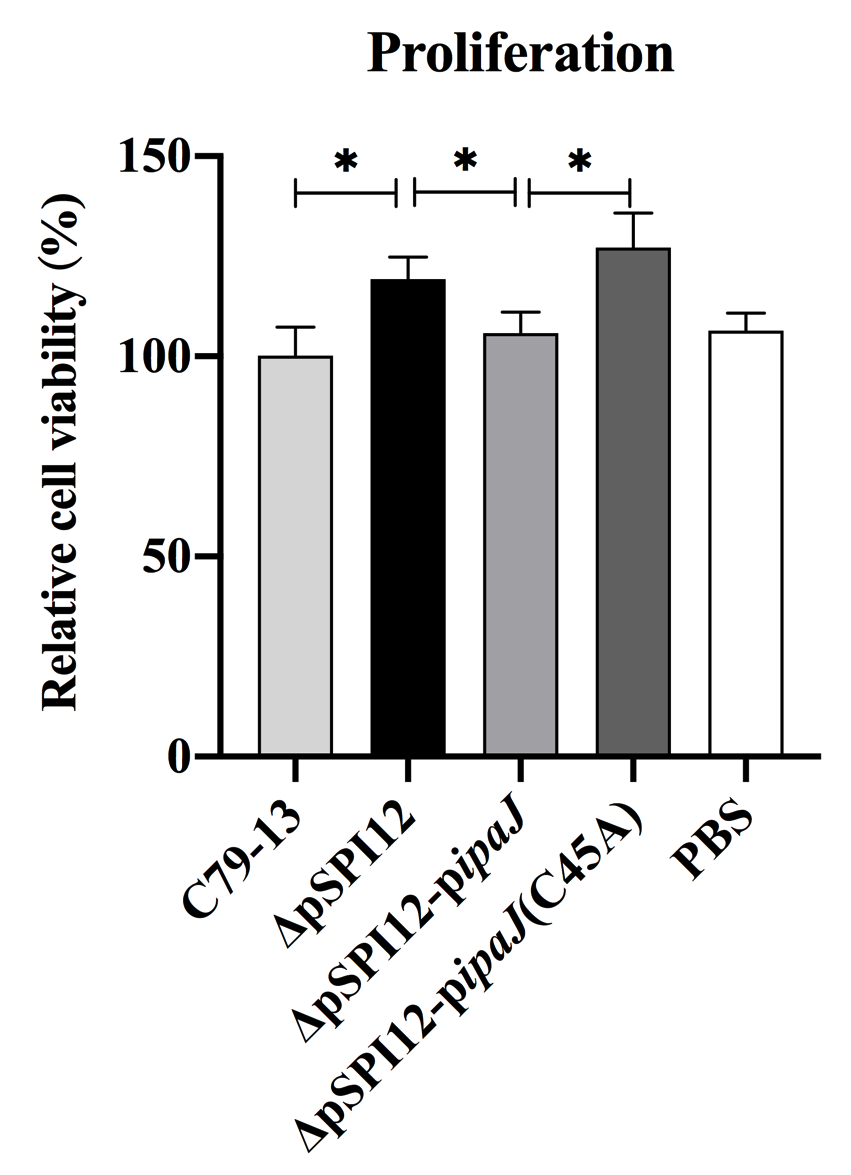

Supplement: S7 Fig — Cell viability was determined using CCK8 assay. The number of cells infected with the WT strain was set at 100%. *: p < 0.05. (TIFF) [file ppat.1011005.s007.tiff]
