# Supplementary material for: The Salmonella T3SS1 effector IpaJ is regulated by ItrA and inhibits the MAPK signaling pathway
Source: PLoS Pathog. 2022 Dec 7;18(12):e1011005. doi: 10.1371/journal.ppat.1011005 (PMC9728880; doi:10.1371/journal.ppat.1011005)
Supplement: S1 Table — (DOCX) [file ppat.1011005.s008.docx]

**S1 Table. *Salmonella* strains and plasmids used in this study**

| **Strains or plasmids** | **Genotype** | **Reference** |
| --- | --- | --- |
| *Salmonella* |  |  |
| C79-13 | *S*. Pullorum wild type | Our laboratory |
| C79-13-P*ipaJ-Cm* | C79-13::Cm-CAT | This study |
| C79-13-Δ*ndh* | *ndh*::FRT mutant | This study |
| C79-13-Δ*hilC* | *hilC*::FRT mutant | This study |
| C79-13-Δ*ramA* | *ramA*::FRT mutant | This study |
| C79-13-Δ*acrA* | *acrA*::FRT mutant | This study |
| C79-13-Δ*acrB* | *acrB*::FRT mutant | This study |
| C79-13-Δ*rcsC* | *rcsC*::FRT mutant | This study |
| C79-13-Δ*yebC* | *yebC*::FRT mutant | This study |
| C79-13-Δ*narX* | *narX*::FRT mutant | This study |
| C79-13-Δ*mntR* | *mntR*::FRT mutant | This study |
| C79-13-Δ*argR* | *argR*::FRT mutant | This study |
| C79-13-Δ*hilA* | *hilA*::FRT mutant | This study |
| C79-13-Δ*ssrB* | *ssrB*::FRT mutant | This study |
| C79-13-Δ*hilD* | *hilD*::FRT mutant | This study |
| ΔpSPI12 | *ipaJ*-deleted mutant | [1] |
| ΔpSPI12-p*ipaJ* | ΔpSPI12::pBR322-*ipaJ* | [1] |
| P125109 | *S*. Enteritidis strain | Our laboratory |
| P125109-p*ipaJ* | *S*. Enteritidis strain carrying pBR322-*ipaJ* | [1] |
| ΔSPI-1 | SPI-1-deleted mutant | This study |
| ΔSPI-2 | SPI-2-deleted mutant | This study |
| ΔSPI-1ΔSPI-2 | SPI-1- and SPI-2-deleted mutant | This study |
| ΔSPI-19 | SPI-19-deleted mutant | This study |
| C79-13-Δ*phoP* | *phoP*::FRT mutant | This study |
| C79-13-Δ*SPN0465* | *SPN0465*::FRT mutant | This study |
| C79-13-Δ*ssrB* | *ssrB*::FRT mutant | This study |
| C79-13-Δ*SPN1235* | *SPN1235*::FRT mutant | This study |
| C79-13-Δ*glcR* | *glcR*::FRT mutant | This study |
| C79-13-Δ*nagC* | *nagC*::FRT mutant | This study |
| C79-13-Δ*ompR* | *ompR*::FRT mutant | This study |
| C79-13-Δ*SPN2262* | *SPN2262*::FRT mutant | This study |
| C79-13-Δ*yiaJ* | *yiaJ* ::FRT mutant | This study |
| C79-13-Δ*SPN3059* | *SPN3059*::FRT mutant | This study |
| C79-13-Δ*treR* | *treR*::FRT mutant | This study |
| C79-13-Δ*lrp* | *lrp*::FRT mutant | This study |
| C79-13-Δ*mraZ* | *mraZ*::FRT mutant | This study |
| C79-13-Δ*SPN3597* | *SPN3597*::FRT mutant | This study |
| C79-13-Δ*yihW* | *yihW*::FRT mutant | This study |
| C79-13-Δ*SPN3824* | *SPN3824*::FRT mutant | This study |
| C79-13-Δ*nhaR* | *nhaR*::FRT mutant | This study |
| C79-13-Δ*SPN4408* | *SPN4408*::FRT mutant | This study |
| C79-13-Δ*aaeR* | *aaeR*::FRT mutant | This study |
| C79-13-Δ*itrA* | Δ*itrA* | This study |
| C79-13-Δ*itrA-*P*itrA* | Δ*itrA*::pBR322-*itrA* | This study |
| WT-pCX340-*ipaJ* | C79-13::pCX340-*ipaJ* | This study |
| ΔSPI-1- pCX340-*ipaJ* | ΔSPI-1::pCX340-*ipaJ* | This study |
| ΔSPI-2- pCX340-*ipaJ* | ΔSPI-2::pCX340-*ipaJ* | This study |
| WT-pCX340 | C79-13::pCX340 | This study |
| Δ*hilA-*pBAD33-*ipaJ* | C79-13-Δ*hilA*::pBAD33-*ipaJ* | This study |
| ΔSPI-1*-*pBAD33-*ipaJ* | ΔSPI-1::pBAD33-*ipaJ* | This study |
| *Escherichia coli* |  |  |
| DH5α λpir | λpir/φ80*lac*ZΔM15 Δ(*lac*ZYA-*arg*F)U169 *rec*A1 *hsd*R17 *deo*R *thi*1 *sup*E44 *gyr*A96 *rel*A1 | Life Technologies |
| χ7213 | *thi-1 thr-1 leuB6 glnV44 fhuA21 lacY1 recA1 RP4-2-Tc::Mu lpir* Δ*asdA4 Dzhf-2::Tn10*; donor strain for conjugation | [1] |
| BL21(DE3) | F^–^ *ompT gal dcm lon hsdSB* (rB^–^ mB^–^) λ(DE3); host for gene expression | TAKARA |
| Plasmids |  |  |
| pDM4 | Suicide plasmid with an R6K origin, *sacBR* and Cm^r^ | [1] |
| pSC189 | Plasmid carrying the mariner transposon, R6K *ori*; Amp^R^ and Km^R^ | [2] |
| pColdⅠ | cold-shock expression vector, Amp^r^ | TAKARA |
| pColdⅠ-*itrA* | pColdⅠ with *itrA*, Amp^r^ | This study |
| pBAD33-*ipaJ* | pBAD33 derivative containing *ipaJ* orf, Cm^r^ | This study |
| pCX340-*ipaJ* | pCX340 derivative containing *ipaJ* orf, Tet^r^ | This study |

**References**

1. Yin C, Xu L, Li Y, Liu Z, Gu D, Li Q, et al. Construction of pSPI12-cured *Salmonella enterica* serovar Pullorum and identification of IpaJ as an immune response modulator. Avian Pathol. 2018;47(4):410-7.

2. Akbar S, Schechter LM, Lostroh CP, Lee CA. AraC/XylS family members, HilD and HilC, directly activate virulence gene expression independently of HilA in *Salmonella typhimurium*. Mol Microbiol. 2003;47(3):715-28.
