# Supplementary material for: The Salmonella T3SS1 effector IpaJ is regulated by ItrA and inhibits the MAPK signaling pathway
Source: PLoS Pathog. 2022 Dec 7;18(12):e1011005. doi: 10.1371/journal.ppat.1011005 (PMC9728880; doi:10.1371/journal.ppat.1011005)
Supplement: S2 Table — (DOCX) [file ppat.1011005.s009.docx]

**Table S2. Primers used in this study**

| **Name** | **Sequence (5’ - 3’)** |
| --- | --- |
| **C79-13-P*ipaJ-Cm* mutant strains construction** | |
| P*ipaJ*-F | GAGCGGATAACAATTTGTGGAATCCCGGGAGACGCACTATCAGGGGAAGG |
| P*ipaJ*-R | CCAGTGATTTTTTTCTCCATACGTGGTTTAACATAATCCCTT |
| PCm-F | AAGGGATTATGTTAAACCACGTATGGAGAAAAAAATCACTGG |
| PCm-R | GAAAAGTGCCACCTGACGTCTCATCGCAGTACTGTTGTAT |
| PAmp-F | ATACAACAGTACTGCGATGAGACGTCAGGTGGCACTTTTC |
| PAmp-R | CGCAACTCACCTTACAACACTTACCAATGCTTAATCAGTGAGGC |
| PDown-F | GCCTCACTGATTAAGCATTGGTAACATTGTTATTGTGTTGTAAGGTGAG |
| PDown-R | AGCGGAGTGTATATCAAGCTTATCGATACCGATACCAGGCGTTTCCCC |
| **Tn-seq mutant strains construction** | |
| RedacrB-F | AAGCGGTGATCAACCTGCTCAGCCCAGGTCTTAACTTAAACAGGAGCCGTTAAGACTGTGTAGGCTGGAGCTGCTTCG |
| RedacrB-R | TTTTATGATTTTTGCGTAAAAAAGGCCGCTTGCGCGGCCTTATCAACAGTGAGCAAACATATGAATATCCTCCTTAG |
| acrB-InF | GGAACCTTTGCCGTGCTT |
| acrB-InR | GGTGTAGTGATGCGTGCTCTTAT |
| acrB-OutF | GTCGTATTGGTAAGTCGTCCGTA |
| acrB-OutR | GGTGTCGTCCAGGTATTCATCT |
| Redndh-F | AAGGCTATACTAATAACTTTTAATTAACAATTGGTTAATAAATTTAAGGGGGTCACGTGTGTAGGCTGGAGCTGCTTCG |
| Redndh-R | GAAGCAAGAGATGGAGAAGCGCTCACCGGCGCGAGCAGCCGGTGAGGCGCAGAGAAACATATGAATATCCTCCTTAG |
| ndh-InF | ATGGCGACGCAGTTAGGC |
| ndh-InR | CAACGATGGCGATATTGACC |
| ndh-OutF | GCAGTTGAATGTGGACGAGC |
| ndh-OutR | ATTTGCGTTCCGGGGTCT |
| RedacrA -F | GGGTTTAAGGACCTTTGACCATTGACCAATTTGAAATCGGACACTCGAGGTTTACATTGTGTAGGCTGGAGCTGCTTCG |
| RedacrA -R | GCAAATATAGGGCGATCGATAAAGAAATTAGGCATGTCTTAACGGCTCCTGTTTAAGCATATGAATATCCTCCTTAG |
| acrA-InF | CGGGTCGTACCGTTGCTT |
| acrA-InR | CCGGACTGCGGGAATTTGA |
| acrA-OutF | AACCATTTCTCCGACAAACTCA |
| acrA-OutR | CTTTTACCGGCGGCGTAC |
| RedramA-F | TATTATGTCATTCGCTTTATCTGGCGGCGCTGGTTTTCGCTGGCCGATTAAACATTTGTGTAGGCTGGAGCTGCTTCG |
| RedramA-R | TGCCGTAAATGTGCGGTGCGGGAGCCGCTGACGAGTTTGATAGAGGGGAGAGCACGCATATGAATATCCTCCTTAG |
| ramA-InF | TTGCTGCGAATCAAAACCA |
| ramA-InR | TGACCATTTCCGCTCAGGT |
| ramA-OutF | TAAACCGCAGGAACAAGACTATC |
| ramA-OutR | AACACCATCAAAACGGAACG |
| RedmntR-F | AATGATCAACAACTGAAAAAAACGGTCACGCCGCAGCGCCCGTAAAAAGGCCAGATTGTGTAGGCTGGAGCTGCTTCG |
| RedmntR-R | ACAATGAGCGCTCTGTCAGCTGACGCGTTAACGCGTCACAGAAACGAGGAAGCAAACATATGAATATCCTCCTTAG |
| mntR-InF | TCCATCCCTTCCGCATCA |
| mntR-InR | GCAGGTACGCCAACAACAAA |
| mntR-OutF | CACCAACACCAGTCCCCATA |
| mntR-OutR | CGCGGTATAAAGCAGGCTAA |
| RedyebC-F | GAAGAGTTTGTCATTAACGTCGCCTGAAAAGGCGCGCTTTTTTTGAGGAAATATTTTGTGTAGGCTGGAGCTGCTTCG |
| RedyebC-R | ATACCGAGAATAATCGACACGCTATCCCCCCTGCCCGCCGGCGTTTAACAGCACCACATATGAATATCCTCCTTAG |
| yebC-InF | TCACTAAAATTATTCGTGAACTGGTA |
| yebC-InR | CGCTTTGGTTGACGGGAT |
| yebC-OutF | GGAGTTGGGGATTCAGGTTG |
| yebC-OutR | CGGTAGGCGATATTGAGACATT |
| RednarX-F | GCCCCTCTTTTTTGCTACGTTTTTTCGGCGACATTACCCCGAAGAAAGAAGGTAACTGTGTAGGCTGGAGCTGCTTCG |
| RednarX-R | ACACCCGTGCGTAGCATCGGATGGTCGTCGATTAACAGGATGGTTGCCGGTTCCTGACATATGAATATCCTCCTTAG |
| narX-InF | CGACGCTGCCCTATGGAC |
| narX-InR | CCTGGGTTTCTGTGAAGTTTGT |
| narX-OutF | GCAAGGAAATAATAATGAAAGTGCT |
| narX-OutR | GGTGGTGGTTGAGGATGAAAA |
| RedrcsC-F | ATTTACCGCTACCTTAGTCACACTCTATTTACATCCTGAGGCGGAGCTTCGCCCCTTGTGTAGGCTGGAGCTGCTTCG |
| RedrcsC-R | TACCAGTCGACTCATCTTTTGCAGGCCGGACAGGCGACGCCGCCATCCGGCATTTTCATATGAATATCCTCCTTAG |
| rcsC-InF | CAACCGTAAAATCGTCGCC |
| rcsC-InR | CTGAGATAATCCCCATCACAGC |
| rcsC-OutF | CTGGCGGGAGACGATAAAG |
| rcsC-OutR | TCCATGCCGGGAGATAAATAC |
| RedhilC-F | TTATTTGTTTATAAGAATATAACGATTTTGAGTTCCTTATAGCACACAGGATAAAATTGTGTAGGCTGGAGCTGCTTCG |
| RedhilC-R | AAAATATTTGGTATCTGTAACGCAAACAGATAGTAACGTTTAAAATAATTTCACAAACATATGAATATCCTCCTTAG |
| hilC-InF | TGAGCGAAATAGAAGGGCATA |
| hilC-InR | AATAGCGATAAAATAAGAAGGCGTA |
| hilC-OutF | GGTGATTCGGCTTTTGTTTCT |
| hilC-OutR | AGCGCAGGATAGTTCGTCTTT |
| RedargR-F | GGCGAGGGGAGAGGACAAAAAGGTGCCTCCGCCGTACGACGACGGAGGAAGTGGGATGTGTAGGCTGGAGCTGCTTCG |
| RedargR-R | AAAATTCATCTGTATGCACAATAATGTTGTTTCTACCGCCATATTACGGGTGACTTCATATGAATATCCTCCTTAG |
| argR-InF | TGGCAGGCGTAGTGAAGATAG |
| argR-InR | CGAAGCTCGGCTAAACAAAA |
| argR-OutF | CATAGGCGATGAGCCGATAG |
| argR-OutR | CGTCTTCACCGGAGAAACCT |
| Red*hilA*-F | GATTCTATCCTAACGACTTGTATTAGTTATTATAACTTTTCACCCTGTAATGTGTAGGCTGGAGCTGCTTCG |
| Red*hilA*-R | CAACCAGATTACGATGATAAAAAAATAATGCATATCTCCTCTCTCAGATTCATATGAATATCCTCCTTAG |
| *hilA*-InF | CCGCAACCTACGACTCATACA |
| *hilA*-InR | TTTAGCTCGCTAATCTGCTTTGT |
| *hilA*-OutF | TAAACCCGAGCCCGTAGAA |
| *hilA*-OutR | AGCCTGAAGCCAGCAATCA |
| Red*hilD*-F | TTTTTCAGTAGGATACCAGTAAGGAACATTAAAATAACATCAACAAAGGGTGTGTAGGCTGGAGCTGCTTCG |
| Red*hilD*-R | TATTACAATTTTAATAAAAATCTTTACTTAAGTGACAGATACAAAAAATGCATATGAATATCCTCCTTAG |
| *hilD*-InF | CAGACTCAGCAGGTTACCATCAA |
| *hilD*-InR | CAGGAACAGCAGAAAATAAGGAAT |
| *hilD*-OutF | AGCAGCAGATTACCGCACAG |
| *hilD*-OutR | TAAGGCAATATCGTTTTACCAGTC |
| Red*ssrB*-F | TATGATCTTCAAAAACTACACCATTACTTAATATTATCTTAATTTTCGCGAGGGCATGTGTAGGCTGGAGCTGCTTCG |
| Red*ssrB*-R | GTGGCGTAAGGCTCATCAAAATATGACCAATGCTTAATACCATCGGACGCCCCTGGCATATGAATATCCTCCTTAG |
| *ssrB*-InF | ACGAGCCTGACATACTTATCCTTG |
| *ssrB*-InR | CCTCATTCTTCGGGCACAGT |
| *ssrB*-OutF | CGCTGGAGGAACTAACCGAC |
| *ssrB*-OutR | AGTGATAAATGGTGCGGCATA |
| **DNA-pull down (DNA-biotin)** | |
| P*ipaJ*-biotin-F | CACTTGAGCGAGCGGACAGAA |
| P*ipaJ*-biotin-R | ACACCTCATACGTGGTTTAACATAAT |
| D*ipaJ*-biotin-F | ATGTGATTAATCCATCTGCTC |
| D*ipaJ*-biotin-R | TATGTTCGATTCCAAGCTCTT |
| **DNA-pull down mutant strains construction** | |
| RednagC-F | ATTATCAAGACCATCGTTAATGGTGACGAGGTCGTTGACTTGAGTAAGTGAGAATTGTGTAGGCTGGAGCTGCTTCG |
| RednagC-R | TGATGGTCATGAACTACCCAGGGAATTTGTGAATAATCCGATACTATAAGAGCGCACATATGAATATCCTCCTTAG |
| nagCIN-F | CCAACGGACGCATCTTCAT |
| nagCIN-R | CGATAGCGGGTAACAGGACTT |
| nagCOut-F | GAGCAGTTCATTTTCGCAGGTA |
| nagCOut-R | GCCTTCGCCCACGACATA |
| RedSPN3597-F | CTGAGAGCATGGCCGTATCGATCAAACTGGCAATGCAACTGGCATAATTCCCTGTTTGTGTAGGCTGGAGCTGCTTCG |
| RedSPN3597-R | ACGGCCGCAATATTTCGGCGGTCTGATTCACCACTGACAGCGCCTGACGGCGCTGCATATGAATATCCTCCTTAG |
| SPN3597IN-F | CGCCAGCCACTATCCGTC |
| SPN3597IN-R | GCATTTCGCCAGTTCCATC |
| SPN3597Out-F | TGGACGGGAAAAGCAAAAT |
| SPN3597Out-R | GTGGATGTAAACGTGTATCAGGAA |
| RedSPN4408-F | CGTTGCCTTACGCATCCAGTTACCATGCGCCAAAGGTTGACGGCGGCGAAAGATATGTGTAGGCTGGAGCTGCTTCG |
| RedSPN4408-R | GCCCTCAGTTAGCCGATGCATTTAGTCTACGGCAATACGCTACGGGCGGATAGCGCCATATGAATATCCTCCTTAG |
| SPN4408IN-F | GAAATGGTCAGTGAACGGGATA |
| SPN4408IN-R | GCCAGCGCCAATAAAACAG |
| SPN4408Out-F | GGTATCGTCCATGCGTTGG |
| SPN4408Out-R | CGCTTCCGTATCGGGTTTA |
| RedSPN1235-F | GGATTTGTTTACACTACCCTCAAGTGGAGCAATTGCTGTCTACATAGGTATGTGCTGTGTAGGCTGGAGCTGCTTCG |
| RedSPN1235-R | TGGCGGCAGGCCCGGTTAATCGCTGACAAAAATGTCATCCGCCTGAAAATAGCCGCATATGAATATCCTCCTTAG |
| SPN1235IN-F | TCGTGAGGCGATTATTATGCTT |
| SPN1235IN-R | CCGCGATGATATTGTGGAAC |
| SPN1235Out-F | AGTCTGGTGGGGAAGGACTG |
| SPN1235Out-R | CGGCATTGGCGTAGAGTTT |
| RedSPN0465-F | CATAGTCTGTGAGATACTCAACGTTTATCATGACGTCGAAAACAGAGGTGTAATCTGTGTAGGCTGGAGCTGCTTCG |
| RedSPN0465-R | CGTTTTTTTTGGCATAATCCAGGCGAAAACAATCCAGGCAAGGATAATCTATCGACATATGAATATCCTCCTTAG |
| SPN0465IN-F | CTAAAACTCCTGAAAGACCGAAAC |
| SPN0465IN-R | CCACCAATAACGCCACCTC |
| SPN0465Out-F | ACAATCCATAAGCAATACATTACACC |
| SPN0465Out-R | TGGCCTCTAACGCAGCAA |
| RedtreR-F | GCTTGAGTAAAGTTACTCTTTTTATCACCATCAGCGTCACGCCACGGGAAAACAGGTGTGTAGGCTGGAGCTGCTTCG |
| RedtreR-R | GAAACGCAGCGACGATCACAAATTACTCGTGGTATTCCGTTTAATCAGTCACTCACATATGAATATCCTCCTTAG |
| treRIN-F | GGGACAAAGCGATAAAGTGGT |
| treRIN-R | GCCCTCGTCGTCGTAACAG |
| treROut-F | AGAGGGGTGGGTTCAGGG |
| treROut-R | AGAGGAGGTAAGTTATGGGTTGG |
| RedyiaJ-F | CACTACGCAAACGGACAAATTTTGTCAGATGCCTGATAAAACCTGGCGAGCTGTCTGTGTAGGCTGGAGCTGCTTCG |
| RedyiaJ-R | ACTGCAACACACTTTGAGCTACCATCTGATCGCCCAAGAAAGGAGAGAGTTTTCTGCATATGAATATCCTCCTTAG |
| yiaJIN-F | GCAACGGTTTGAGCAGATTTT |
| yiaJIN-R | GGATTATGTGGCGTCTTACTGG |
| yiaJOut-F | AAAGTCCGCCGTCGTCAC |
| yiaJOut-R | CATAATGCGAAATGTAGTTCCAAA |
| RedaaeR-F | GTCAGCCTCGGGTGGCATCTGGCCTACAATCGTCTTCAGCAATTACCCGGTGTTTTGTGTAGGCTGGAGCTGCTTCG |
| RedaaeR-R | TGTTATGCTGTTATCTATATTATGTGATCTAAATCACTTTTAAGCCAGAGTGAATACATATGAATATCCTCCTTAG |
| aaeRIN-F | TCGGACTGATAGCGAGGCA |
| aaeRIN-R | GACGGACTGGACGTGGTGA |
| aaeROut-F | AAAGTCCGCCGTCGTCAC |
| aaeROut-R | ACAAGCATCCGACGCACC |
| RedyihW-F | CTTTCTTGTCACTTTTTGTATAATATGAGCAGTAGGAAGCTTTTAGAGGAATGCTCTGTGTAGGCTGGAGCTGCTTCG |
| RedyihW-R | CCGTTTTATTGGCTGGATAAAGCGCTGACGCGACCATCCGGCACAAGGGCGCTTGCATATGAATATCCTCCTTAG |
| yihWIN-F | AGCAGGGGCTGATTACGC |
| yihWIN-R | CGCCAGGCACCATCACTT |
| yihWOut-F | GGCTGCAACTGGTTGGAAA |
| yihWOut-R | GAATAACTGTAGCGGGAAAGGA |
| RedompR-F | ACGATGAGCAACAGCGTGCGGGCAAATGAACTTCGCGGCGAGAAGCGCATTCGCCTGTGTAGGCTGGAGCTGCTTCG |
| RedompR-R | GAGCTTTTTTAAGAATACACACTTACATTTGTTGCGAACCTTTGGGAGTACAGACACATATGAATATCCTCCTTAG |
| ompRIN-F | TTCAGTACCGCAAACTCCCC |
| ompRIN-R | GCCGATGACTACATTCCTAAACC |
| ompROut-F | CAGCGGCTTCGTTGGTGT |
| ompROut-R | AGGGGCGTTTTCATCTCGT |
| RedglcR-F | AACAGTTTACGCAACTGGTACGCAAGATGATGACGTAAGCGGCTTGCGCCGTTTTGTGTGTAGGCTGGAGCTGCTTCG |
| RedglcR-R | CGCTGTGACGAGAAATCGCGTAAAATCGCGACAGGACTAAAGAAAGGAGCAGACGCATATGAATATCCTCCTTAG |
| glcRIN-F | AATCATCAACCCACGCAAAA |
| glcRIN-R | ACCCTCGCATCAGATCAAAAC |
| glcROut-F | ACGATCCGCTGGATAAACG |
| glcROut-R | GAGCCAAGCCCCACAATG |
| RedSPN3824-F | ACAGCGGGTAATGAGCGCTGTGCGTAAACTCCTTTCCGCGCCGCAACATACCCACCTGTGTAGGCTGGAGCTGCTTCG |
| RedSPN3824-R | GACATCGGTTTTCGACGTTACTGTTTCCTCTGTTTGCGATCGGCAGGGGATTATCCATATGAATATCCTCCTTAG |
| SPN3824IN-F | GAAACAGGCGGCAGAAATAGT |
| SPN3824IN-R | CGGCAGCCAAATCCAGAC |
| SPN3824Out-F | GGGCGTGGAGCGATTTAC |
| SPN3824Out-R | TCACCAACCCGCAGTTCA |
| RedssrB-F | TGGCGTAAGGCTCATCAAAATATGACCAATGCTTAATACCATCGGACGCCCCTGGTGTGTAGGCTGGAGCTGCTTCG |
| RedssrB-R | CTTCAAAAACTACACCATTACTTAATATTATCTTAATTTTCGCGAGGGCAGCAAACATATGAATATCCTCCTTAG |
| ssrBIN-F | ACCTCATTCTTCGGGCACA |
| ssrBIN-R | AAACAGTAGCAGTAAACAAGCGTTA |
| ssrBOut-F | GCATTGATACCGCATAGTCTGG |
| ssrBOut-R | CGCTGGAGGAACTAACCGAC |
| RedphoP-F | AGCAAAAAACGAACCCGCAGCGACAGCGGCAGAAAATGGCGAGCAAATTTATTCATGTGTAGGCTGGAGCTGCTTCG |
| RedphoP-R | CAAAGCACCATAATCAACGCTAGACTGTTCTTATTGTTAACACAAGGGAGAAGAGCATATGAATATCCTCCTTAG |
| phoPIN-F | CCTTGCTGACCACTTTACCG |
| phoPIN-R | TGACGAAGCCATTCCACATC |
| phoPOut-F | GCAGCAAACGAAAGGTGGT |
| phoPOut-R | AAGCGTGTTGATGCCGAAG |
| RednhaR-F | AATGATTCCGGCTGCGGGTTAATACCGCAGCCGGAAGTATTACGGTAGGTACGTTGTGTAGGCTGGAGCTGCTTCG |
| RednhaR-R | GAAAGGGGGAGCGATGTATCGCTCCCTTGCGACATTGAACAGGGAGAGAAATGAGCCATATGAATATCCTCCTTAG |
| nhaRIN-F | CGACGCCGACATCAAACA |
| nhaRIN-R | TGACGCCGCAAACCATTA |
| nhaROut-F | AGGGGAAACATGGACGGAT |
| nhaROut-R | TGTGGCATTGGCTTTACGAT |
| RedSPN2262-F | ACCGGCTGCGTAAGTACCTGGCGCGTTTTGGGCTGACGTGGGACGTTTTACAGAATGTGTAGGCTGGAGCTGCTTCG |
| RedSPN2262-R | CGGGTAGGGCGAATGCCGCTATCCGGCGCAAGGTTGGTTACTTTCAGAGATTATTCCATATGAATATCCTCCTTAG |
| SPN2262IN-F | CGGCAGGGTATCGGTGTAG |
| SPN2262IN-R | TATCGCCCAGTTCCGTCTC |
| SPN2262Out-F | CTGGATCTGTTTGACCGTATGC |
| SPN2262Out-R | GGTTTAGCGATGGCCTTTGT |
| RedSPN3059-F | AATGTGATGAATTTCGCCAGTCCGTTGATATTAATAATAAAGATAAGGTGCATTTTGTGTAGGCTGGAGCTGCTTCG |
| RedSPN3059-R | GCTCTGTTTGGGGATCAAGCGAATTAATGCATGATTTACTCATCGCAAACGGTTCCATATGAATATCCTCCTTAG |
| SPN3059IN-F | ATCAGAAATACGAAGGGACAAAAG |
| SPN3059IN-R | GACCCAGACAATACTGCTACCG |
| SPN3059Out-F | ACATTGCTGAATATGGCGGTAC |
| SPN3059Out-R | GCTGCGGGTTGGCTTTTA |
| Redlrp-F | AGTGATGTGAGTAGAGTCAGGCAGGAGTAGGGAAGGAATACAGAGAGACAATAATATGTGTAGGCTGGAGCTGCTTCG |
| Redlrp-R | CTGTATGGATTAATAGGAGTGTAATCAAACTACAGCGATTTTGCACCTGTTCCGTGCATATGAATATCCTCCTTAG |
| lrpIN-F | AACGTCGAGCTTTCTAAACGAGTAG |
| lrpIN-R | TGTTTTACCTCTTCCATCACTACG |
| lrpOut-F | GCGAGGCAACGGTCTTCT |
| lrpOut-R | GCTGCTTAACTTCGTCAATGTG |
| RedmraZ-F | AAACTCCTTCGAGTGGGAATTTGTGGGATAAAGTGGTAAGAAGGGGTGAGACTGGCTGTGTAGGCTGGAGCTGCTTCG |
| RedmraZ-R | GCCCATTAACGGCTTCATCCAACAGTACCGTAGTGTGTTTAAAATTTTCCATCATCCATATGAATATCCTCCTTAG |
| mraZIN-F | TGCCTGCTGCTTTACCCC |
| mraZIN-R | CATCACTTCTTTCGTCAGTCCG |
| mraZOut-F | TTACTTAGGTAAGCGTTGTGACTTG |
| mraZOut-R | AATGATGGAGAAGCGAGGATC |
| P*itrA*-F | GTTTGACAGCTTATCATCGATGTATGATATGGTGGTGGCGA |
| P*itrA*-R | ACGATGCGTCCGGCGTAGATCACTCCGGCTGCACCAG |
| **Protein expression** | |
| pd*itrA*-F | CCCTCGAGGGATCCGAATTCATGAAAGGACAACACCGTCTGG |
| Pd*itrA*-R | TATCTAGACTGCAGGTCGACTCACTCCGGCTGCACCAG |
| **FRET** |  |
| pCX340-*ipaJ*-F | AATAAGGAGGAATAACATATGATGAGGTGTTATGTGATTAATC |
| pCX340-*ipaJ*-R | CGAATTCTCCGCGGAGGTACCTCAAGCTGACAAGACAATAGAA |
| **EMSA** | |
| P*ipaJ*-EMSA-F | CACTTGAGCGAGCGGACAGAA |
| P*ipaJ*-EMSA-R | ACACCTCATACGTGGTTTAACATAAT |
| D*ipaJ*-EMSA-F | ATGTGATTAATCCATCTGCTC |
| D*ipaJ*-EMSA-R | TATGTTCGATTCCAAGCTCTT |
| P-240~-141-F | TGCCTGCAGGTCGACGATGATGGTCCTTCGTTGTCGC |
| P-240~-141-R | CCTATGCAACCAGATGGTGC |
| P-190~-91-F | TGCCTGCAGGTCGACGATTCGGTGGATTTTGACGGG |
| P-190~-91-R | ACCCGAAAAACTGCCGCG |
| P-140~-41-F | TGCCTGCAGGTCGACGATTATTCATACGGTTAAAATTTATCAG |
| P-140~-41-R | ACGCGTTCATCGCGATCA |
| P-90~+10-F | TGCCTGCAGGTCGACGATGGTTTGTTGCCATTTTTACCT |
| P-90~+10-R | AACACCTCATACGTGGTTTAACATA |
| FAM | TGCCTGCAGGTCGACGAT |
| **Sequencing** | |
| **Adaptor biosynthesis** | |
| AD_fork truncated NH2 | TACCACGACCA |
| AD_Index Fork R | GTGACTGGAGTTCAGACGTGTGCTCTTCCGATCTGGTCGTGGTAT |
| **1^st^ PCR** | |
| Seq-Out (pSC189) | ACAATTCGTTCAAGCCGAGAT |
| Index ‘R’ primer | GTGACTGGAGTTCAGACGTGTG |
| **2^nd^ PCR** | |
| P5-InvRep-Var1F | AATGATACGGCGACCACCGAGATCTACACTCTTTCCCTACACGACGCTCTTCCGATCTGACTTATCAGCCAACCTGT |
| P5-InvRep-Var2F | AATGATACGGCGACCACCGAGATCTACACTCTTTCCCTACACGACGCTCTTCCGATCTCGACTTATCAGCCAACCTGT |
| P5-InvRep-Var3F | AATGATACGGCGACCACCGAGATCTACACTCTTTCCCTACACGACGCTCTTCCGATCTATGACTTATCAGCCAACCTGT |
| P5-InvRep-Var4F | AATGATACGGCGACCACCGAGATCTACACTCTTTCCCTACACGACGCTCTTCCGATCTTGTCGACTTATCAGCCAACCTGT |
| P5-InvRep-Var5F | AATGATACGGCGACCACCGAGATCTACACTCTTTCCCTACACGACGCTCTTCCGATCTTCGACGACTTATCAGCCAACCTGT |
| P5-InvRep-Var6F | AATGATACGGCGACCACCGAGATCTACACTCTTTCCCTACACGACGCTCTTCCGATCTGCAGCGACGACTTATCAGCCAACCTGT |
| P7-AD001-index-R | CAAGCAGAAGACGGCATACGAGATCGTGATGTGACTGGAGTTCAGACGTGTGCTCTTCCGATC |
