# Supplementary material for: The Salmonella T3SS1 effector IpaJ is regulated by ItrA and inhibits the MAPK signaling pathway
Source: PLoS Pathog. 2022 Dec 7;18(12):e1011005. doi: 10.1371/journal.ppat.1011005 (PMC9728880; doi:10.1371/journal.ppat.1011005)
Supplement: S3 Table — (DOCX) [file ppat.1011005.s010.docx]

**Table S3 Putative activators of *ipaJ* identified by Tn-Seq**

| **CDS** | **Gene** | **Annotation** | **Fold**  **(Output/Input)** | ***p* value** |
| --- | --- | --- | --- | --- |
| EGFFDBLF_04547' | *SPN4547* | hypothetical protein | ∞ | 0.00749 |
| 'EGFFDBLF_03382' | *fabA* | 3-hydroxydecanoyl-[acyl-carrier-protein] dehydratase | 0.105263 | 0.003219 |
| EGFFDBLF_01882' | *arcB* | Multidrug efflux pump subunit AcrB | 0.142246 | 0.000648 |
| 'EGFFDBLF_02578' | *trxC* | Thioredoxin 2 | 0.142857 | 0.018931 |
| 'EGFFDBLF_00370' | *ndh* | NADH dehydrogenase | 0.154632 | 0.000132 |
| 'EGFFDBLF_01881' | *acrA* | Multidrug efflux pump subunit AcrA | 0.162829 | 0.000419 |
| 'EGFFDBLF_00352' | *fabD* | Malonyl CoA-acyl carrier protein transacylase | 0.178571 | 0.028439 |
| 'EGFFDBLF_00640' | *SPN0640* | Electron transport complex subunit RsxE | 0.181818 | 0.033472 |
| 'EGFFDBLF_02925' | *nrdA* | Ribonucleoside-diphosphate reductase 1 subunit alpha | 0.181818 | 0.033472 |
| EGFFDBLF_00011' | *YejM* | Inner membrane protein YejM | 0.199017 | 5.2E-05 |
| 'EGFFDBLF_00676' | *osmV* | Osmoprotectant import ATP-binding protein OsmV | 0.228571 | 0.039214 |
| 'EGFFDBLF_00160' | *rfbH* | GDP-4-keto-6-deoxy-D-mannose 3-dehydratase | 0.236264 | 0.004572 |
| 'EGFFDBLF_00988' | *lptC* | Lipopolysaccharide export system protein LptC | 0.241379 | 0.01536 |
| 'EGFFDBLF_03961' | *lpxM* | Potassium-transporting ATPase KdpC subunit | 0.290503 | 0.004951 |
| 'EGFFDBLF_04673' | *hemE* | Uroporphyrinogen decarboxylase | 0.293103 | 0.048028 |
| EGFFDBLF_01776' | *ramA* | Transcriptional activator RamA | 0.304015 | 0.002373 |
| EGFFDBLF_01011' | *ftsH* | ATP-dependent zinc metalloprotease FtsH | 0.306122 | 0.002443 |
| EGFFDBLF_00563' | *laaA* | L-amino acid amidase | 0.30814 | 0.001836 |
| EGFFDBLF_03863' | *SPN3863* | hypothetical protein | 0.320611 | 0.021621 |
| EGFFDBLF_04587' | *argR* | Transcriptional regulator ArgR | 0.321839 | 0.039671 |
| EGFFDBLF_01812' | *cysS* | Cysteine--tRNA ligase | 0.333333 | 0.021312 |
| EGFFDBLF_03803' | *metJ* | Met repressor | 0.343284 | 0.048265 |
| EGFFDBLF_01130' | *tolC* | Outer membrane protein TolC | 0.347092 | 0.006321 |
| EGFFDBLF_02481' | *coaD* | Phosphopantetheine adenylyltransferase | 0.35 | 0.004357 |
| EGFFDBLF_00159' | *rfbG* | CDP-glucose 4,6-dehydratase | 0.350427 | 0.023565 |
| EGFFDBLF_00106' | *ydcP* | putative protease YdcP | 0.360294 | 0.02201 |
| EGFFDBLF_02479' | *rfaQ* | Lipopolysaccharide core heptosyltransferase RfaQ | 0.362854 | 0.009519 |
| EGFFDBLF_04238' | *corA* | Magnesium transport protein CorA | 0.366279 | 0.000501 |
| EGFFDBLF_02472' | *rfaY* | Lipopolysaccharide core heptose(II) kinase RfaY | 0.371429 | 0.010835 |
| EGFFDBLF_01989' | *hemB* | Delta-aminolevulinic acid dehydratase | 0.372093 | 0.02656 |
| EGFFDBLF_00891' | *lapB* | hypothetical protein | 0.382979 | 0.015844 |
| EGFFDBLF_02815' | *prmB* | 50S ribosomal protein L3 glutamine methyltransferase | 0.383234 | 0.000544 |
| EGFFDBLF_01871' | *ybaB* | Nucleoid-associated protein YbaB | 0.384615 | 0.003812 |
| EGFFDBLF_01940' | *pgpA* | Phosphatidylglycerophosphatase A | 0.384615 | 0.046911 |
| EGFFDBLF_04432' | *mntR* | Transcriptional regulator MntR | 0.392405 | 0.011189 |
| EGFFDBLF_01728' | *citD* | Citrate lyase acyl carrier protein | 0.394737 | 0.002775 |
| EGFFDBLF_01328' | *thyA* | Thymidylate synthase | 0.397959 | 0.047558 |
| EGFFDBLF_01110' | *SPN1110* | Multifunctional CCA protein | 0.402299 | 0.020577 |
| EGFFDBLF_03854' | *ompS* | Outer membrane protein N | 0.408163 | 0.009278 |
| EGFFDBLF_01715' | *SPN1715* | Octanoyl transferase | 0.408284 | 0.003277 |
| EGFFDBLF_01697' | *SPN1697* | hypothetical protein | 0.41295 | 0.001682 |
| EGFFDBLF_00406' | *SPN0406* | Attachment invasion locus protein | 0.414791 | 0.004619 |
| EGFFDBLF_02154' | *SPN2154* | hypothetical protein | 0.414791 | 0.032579 |
| EGFFDBLF_01465' | *prgI* | Protein PrgI | 0.418605 | 0.011768 |
| EGFFDBLF_02866' | *SPN2866* | hypothetical protein | 0.421911 | 0.009278 |
| EGFFDBLF_00411' | *SPN0411* | hypothetical protein | 0.424419 | 0.001116 |
| EGFFDBLF_03157' | *SPN3157* | Phosphatidylserine decarboxylase proenzyme | 0.427184 | 0.034839 |
| EGFFDBLF_03951' | *yebC* | putative transcriptional regulatory protein YebC | 0.427928 | 0.00314 |
| EGFFDBLF_02876' | *nuoE* | NADH-quinone oxidoreductase subunit E | 0.42953 | 0.005578 |
| EGFFDBLF_03987' | *narX* | Nitrate/nitrite sensor protein NarX | 0.430493 | 0.016879 |
| EGFFDBLF_00631' | *slyB* | Outer membrane lipoprotein SlyB | 0.43083 | 4.01E-06 |
| EGFFDBLF_01576' | *SPN1576* | hypothetical protein | 0.436441 | 0.010599 |
| EGFFDBLF_00900' | *topA* | DNA topoisomerase 1 | 0.440217 | 0.005191 |
| EGFFDBLF_01277' | *fldB* | Flavodoxin 2 | 0.440299 | 0.039816 |
| EGFFDBLF_04613' | *SPN4613* | hypothetical protein | 0.445087 | 0.003745 |
| EGFFDBLF_04244' | *SPN4244* | hypothetical protein | 0.449782 | 0.019034 |
| EGFFDBLF_04342' | *dnaN* | Beta sliding clamp | 0.456311 | 0.045862 |
| EGFFDBLF_04713' | *SPN4713* | hypothetical protein | 0.45641 | 0.003835 |
| EGFFDBLF_00154' | *rfbD* | dTDP-4-dehydrorhamnose reductase | 0.456522 | 0.009141 |
| EGFFDBLF_00925' | *cls* | Cardiolipin synthase A | 0.459368 | 0.000215 |
| EGFFDBLF_04213' | *rfaH* | Transcription antitermination protein RfaH | 0.459459 | 0.026843 |
| EGFFDBLF_02880' | *nuoI* | NADH-quinone oxidoreductase subunit I | 0.462121 | 0.033894 |
| EGFFDBLF_04628' | *SPN4628* | Lysozyme RrrD | 0.462366 | 0.003471 |
| EGFFDBLF_00088' | *yehD* | putative fimbrial-like protein YehD | 0.464968 | 0.004252 |
| EGFFDBLF_02641' | *hscA* | Chaperone protein HscA | 0.468085 | 0.039352 |
| EGFFDBLF_01884' | *hha* | Hemolysin expression-modulating protein Hha | 0.469697 | 0.028505 |
| EGFFDBLF_02708' | *mexB* | Multidrug resistance protein MexB | 0.470219 | 0.029358 |
| EGFFDBLF_00890' | *pyrF* | Orotidine 5'-phosphate decarboxylase | 0.470226 | 0.029915 |
| EGFFDBLF_00105' | *SPN0105* | Tir chaperone | 0.471366 | 0.01866 |
| EGFFDBLF_01008' | *greA* | Transcription elongation factor GreA | 0.472103 | 0.027515 |
| EGFFDBLF_02934' | *rcsC* | Sensor histidine kinase RcsC | 0.474227 | 0.042613 |
| EGFFDBLF_02160' | *radC* | hypothetical protein | 0.475162 | 0.002212 |
| EGFFDBLF_00905' | *rluB* | Ribosomal large subunit pseudouridine synthase B | 0.475362 | 0.015342 |
| EGFFDBLF_03581' | *pdhR* | Pyruvate dehydrogenase complex repressor | 0.476744 | 0.002787 |
| EGFFDBLF_04575' | *mreD* | Rod shape-determining protein MreD | 0.476923 | 0.027064 |
| EGFFDBLF_00987' | *lptA* | Lipopolysaccharide export system protein LptA | 0.477612 | 0.016813 |
| EGFFDBLF_00884' | *SPN0884* | Exoribonuclease 2 | 0.477673 | 0.004385 |
| EGFFDBLF_01471' | *hilC* | HTH-type transcriptional regulator SirC/HilC | 0.478814 | 0.012404 |
| EGFFDBLF_03320' | *aat* | Leucyl/phenylalanyl-tRNA--protein transferase | 0.479814 | 0.00134 |
| EGFFDBLF_04288' | *maoP* | hypothetical protein | 0.482972 | 0.008455 |
| EGFFDBLF_03371' | *pncB* | icotinate phosphoribosyltransferase | 0.483209 | 0.00164 |
| EGFFDBLF_02937' | *ompC* | Outer membrane protein C | 0.485981 | 0.04346 |
| EGFFDBLF_02521' | *SPN2521* | hypothetical protein | 0.487179 | 0.036295 |
| EGFFDBLF_01133' | *cpdA* | 3',5'-cyclic adenosine monophosphate phosphodiesterase CpdA | 0.488636 | 0.03772 |
| EGFFDBLF_03780' | *lsrG* | (4S)-4-hydroxy-5-phosphonooxypentane-2,3-dione isomerase | 0.489051 | 0.035168 |
| EGFFDBLF_01745' | *ahpC* | Alkyl hydroperoxide reductase C | 0.490716 | 0.001386 |
| EGFFDBLF_00125' | *asmA* | hypothetical protein | 0.491577 | 0.003044 |
| EGFFDBLF_01909' | *clpX* | ATP-dependent Clp protease ATP-binding subunit ClpX | 0.492401 | 0.005581 |
| EGFFDBLF_04065' | *SPN4065* | hypothetical protein | 0.492857 | 0.029345 |
| EGFFDBLF_01911' | *clpP* | ATP-dependent Clp protease proteolytic subunit | 0.493056 | 0.015485 |
| EGFFDBLF_00834' | *hrpA* | hypothetical protein | 0.493934 | 0.001711 |
| EGFFDBLF_00993' | *mlaE* | putative phospholipid ABC transporter permease protein MlaE | 0.497462 | 0.020627 |
| EGFFDBLF_00643' | *rsxC* | Electron transport complex subunit RsxC | 0.5 | 0.020269 |
| EGFFDBLF_02131' | *SPN2131* | hypothetical protein | 0.5 | 0.045728 |
