# Supplementary material for: The Salmonella T3SS1 effector IpaJ is regulated by ItrA and inhibits the MAPK signaling pathway
Source: PLoS Pathog. 2022 Dec 7;18(12):e1011005. doi: 10.1371/journal.ppat.1011005 (PMC9728880; doi:10.1371/journal.ppat.1011005)
Supplement: S4 Table — (DOCX) [file ppat.1011005.s011.docx]

**Table S4 MS results of proteins screened by DNA pull-down assay**

| **CDS** | **Protein** | **Annotation** | **Coverage(%)** | **Mass** |
| --- | --- | --- | --- | --- |
| EGFFDBLF_01668' | NagC | DNA-binding transcriptional regulator NagC | 45.57 | 44493.9 |
| EGFFDBLF_03324' | Lrp | leucine-responsive transcriptional regulator Lrp | 56.71 | 18856.6 |
| EGFFDBLF_01456' | SipA | SPI-1 type III secretion system effector SipA | 25.99 | 73971.4 |
| EGFFDBLF_03597' | SPN3597 | DeoR/GlpR transcriptional regulator | 40.39 | 27861.9 |
| EGFFDBLF_04408' | SPN4408 | DeoR/GlpR transcriptional regulator | 31.75 | 27701.7 |
| EGFFDBLF_04415' | LclR | glyoxylate bypass operon transcriptional repressor IclR | 30.66 | 29613.7 |
| EGFFDBLF_01235' | SPN1235 | FadR family transcriptional regulator | 37.75 | 29416.3 |
| EGFFDBLF_00465' | SPN0465 | DeoR/GlpR transcriptional regulator | 25.79 | 28425.3 |
| EGFFDBLF_03406' | SopB | SPI-1 type III secretion system effector inositol phosphate phosphatase SopB | 8.2 | 61988.4 |
| EGFFDBLF_01440' | InvG/MxiD | type III secretion system outer membrane ring protein InvG/MxiD | 11.39 | 61742.2 |
| EGFFDBLF_03546' | MraZ | Transcriptional regulator MraZ | 32.24 | 17434.9 |
| EGFFDBLF_00936' | H-NS | DNA-binding transcriptional regulator H-NS | 29.93 | 15570.6 |
| EGFFDBLF_03067' | TreR | HTH-type transcriptional regulator TreR | 17.14 | 34752.7 |
| EGFFDBLF_02420' | YiaJ | IclR family transcriptional regulator YiaJ | 20.3 | 29446.5 |
| EGFFDBLF_03380' | PqiB | intermembrane transport protein PqiB | 9.158 | 60626 |
| EGFFDBLF_04580' | AaeR | HTH-type transcriptional activator AaeR | 16.5 | 34341.5 |
| EGFFDBLF_03720' | YihW | DeoR-type transcriptional regulator YihW | 15.36 | 29044.6 |
| EGFFDBLF_02240' | OmpR | Transcriptional regulatory protein OmpR | 20.08 | 27353.3 |
| EGFFDBLF_02514' | SPN2514 | virulence RhuM family protein | 14.78 | 40070 |
| EGFFDBLF_01471' | HilC | transcriptional regulator HilC | 10.85 | 33854.8 |
| EGFFDBLF_01420' | SPN1420 | DeoR/GlpR transcriptional regulator | 16.14 | 27466.6 |
| EGFFDBLF_03824' | SPN3824 | AraC family transcriptional regulator | 13.78 | 32371.7 |
| EGFFDBLF_00575' | NreC | Oxygen regulatory protein NreC /two component system response regulator | 13.68 | 24354.3 |
| EGFFDBLF_00390' | PhoP | Virulence transcriptional regulatory protein PhoP | 21.43 | 25633.1 |
| EGFFDBLF_04115' | NhaR | Transcriptional activator protein NhaR | 10.1 | 33779.5 |
| EGFFDBLF_02605' | MurR/RpiR | HTH-type transcriptional regulator MurR/RpiR | 14.18 | 30925.4 |
| EGFFDBLF_03212' | PmrA/BasR | two-component system response regulator PmrA/BasR | 12.61 | 25053.3 |
| EGFFDBLF_04645' | SPN4645 | transcriptional regulator/helix-turn-helix domain-containing protein | 30 | 12419.9 |
| EGFFDBLF_01302' | RcnR | Transcriptional repressor RcnR | 28.89 | 10207.8 |
| EGFFDBLF_02774' | SPN2774 | LysR family transcripitonal regulator | 10.2 | 32955.1 |
| EGFFDBLF_02262' | SPN2262 | DeoR/GlpR family transcriptional regulator | 8.73 | 27927.5 |
| EGFFDBLF_04030' | FadR | fatty acid metabolism transcriptional regulator FadR | 8.787 | 26986.4 |
| EGFFDBLF_04432' | MntR | Transcriptional regulator MntR | 16.56 | 17728.1 |
| EGFFDBLF_03526' | AraC | arabinose operon transcriptional regulator AraC | 7.829 | 32070.1 |
| EGFFDBLF_00510' | IF-3 | Translation initiation factor IF-3 | 17.54 | 19667.7 |
| EGFFDBLF_04587' | ArgR | transcriptional regulator ArgR | 8.333 | 17064.5 |
| EGFFDBLF_01023' | IF-2 | Translation initiation factor IF-2 | 2.242 | 97373.5 |
| EGFFDBLF_01964' | PhoB | Phosphate regulon transcriptional regulatory protein PhoB | 12.66 | 26403.2 |
| EGFFDBLF_02450' | GntR | HTH-type transcriptional regulator GntR | 7.485 | 36232.5 |
| EGFFDBLF_01199' | SPN1199 | Hca operon transcriptional activator HcaR/LysR family transcriptional regulator | 5.479 | 31990.9 |
| EGFFDBLF_03059' | SPN3059 | ArgR family transcriptional regulator | 10.49 | 18220.8 |
| EGFFDBLF_01647' | KdpE | two-component system response regulator KdpE | 4.889 | 25313.7 |
| EGFFDBLF_02548' | SPN2548 | DeoR family transcriptional regulator | 7.28 | 28959 |
| EGFFDBLF_03770' | SPN3770 | GntR family transcriptional regulator | 5 | 27009.5 |
| EGFFDBLF_01349' | GcvA | glycine cleavage system transcriptional regulator GcvA | 4.59 | 34404.2 |
| EGFFDBLF_00614' | PurR | HTH-type transcriptional repressor PurR | 3.226 | 38048.2 |
| EGFFDBLF_00887' | SPN0887 | DeoR/GlpR transcriptional regulator | 5 | 26290.9 |
| EGFFDBLF_03138' | NsrR | HTH-type transcriptional repressor NsrR | 4.965 | 15608 |
| EGFFDBLF_01299' | SPN1299 | CaiF/GrlA family transcriptional regulator | 5.882 | 19633.1 |
